# Supplementary figures and images for: Characterization of RNA polymerase II trigger loop mutations using molecular dynamics simulations and machine learning
Source: PLoS Comput Biol. 2023 Mar 22;19(3):e1010999. doi: 10.1371/journal.pcbi.1010999 (PMC10069792; doi:10.1371/journal.pcbi.1010999)

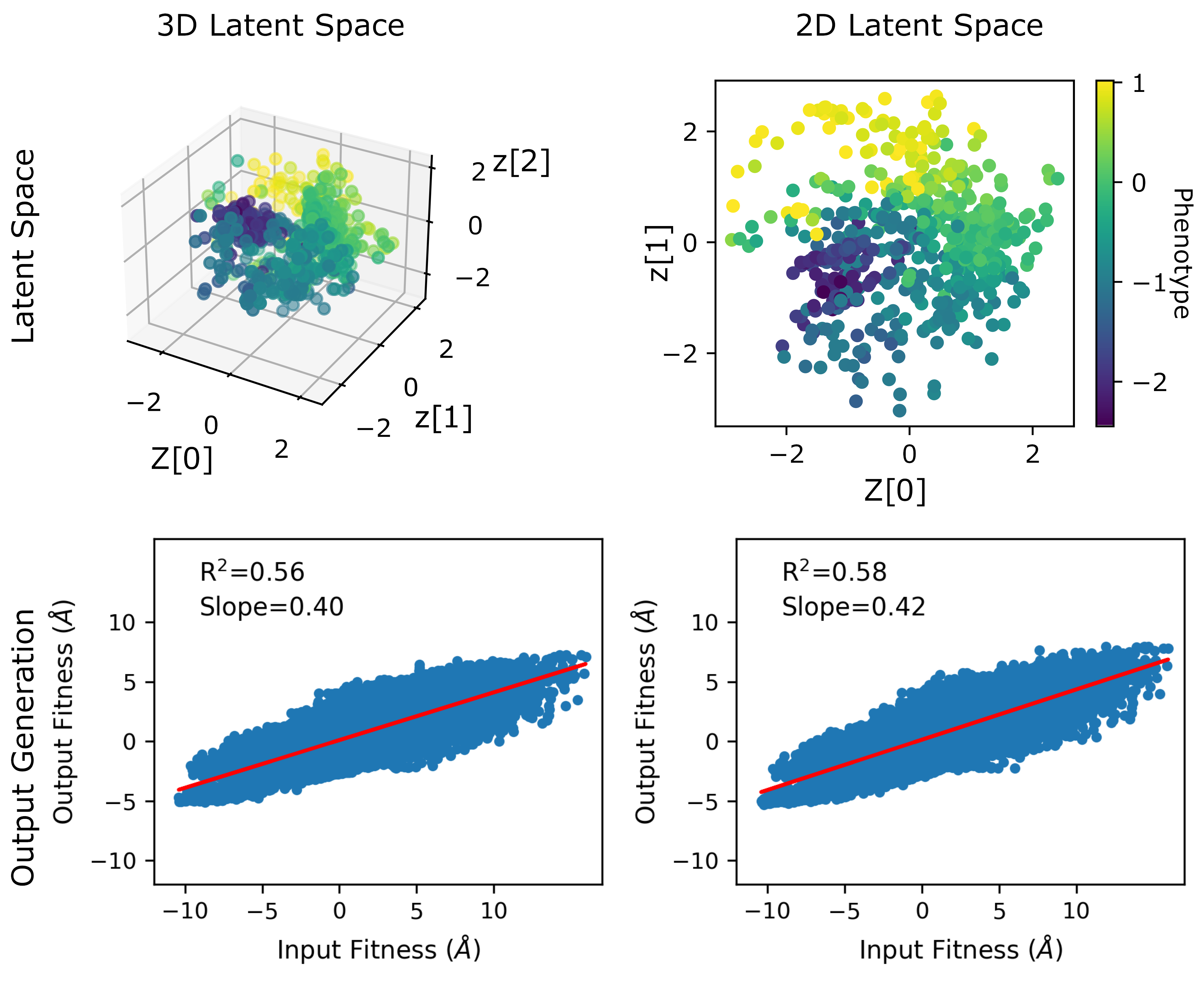

Supplement: S1 Fig — The distribution of mutants on the latent spaces (top) and generative performances (bottom) of the VAE models with 3D (left) and 2D (right) latent spaces using fitness data as the input. (TIF) [file pcbi.1010999.s001.tif]

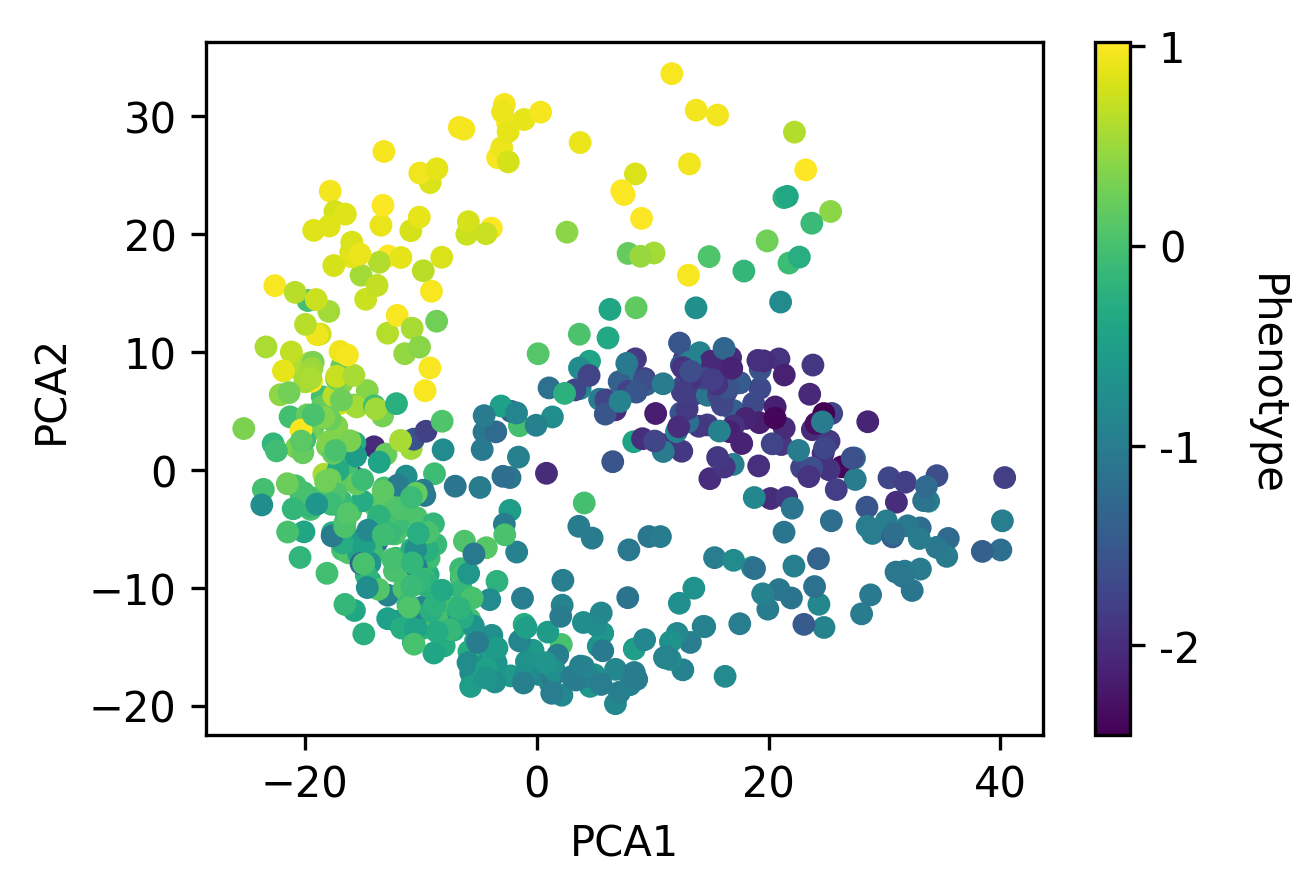

Supplement: S2 Fig — (TIF) [file pcbi.1010999.s002.tif]

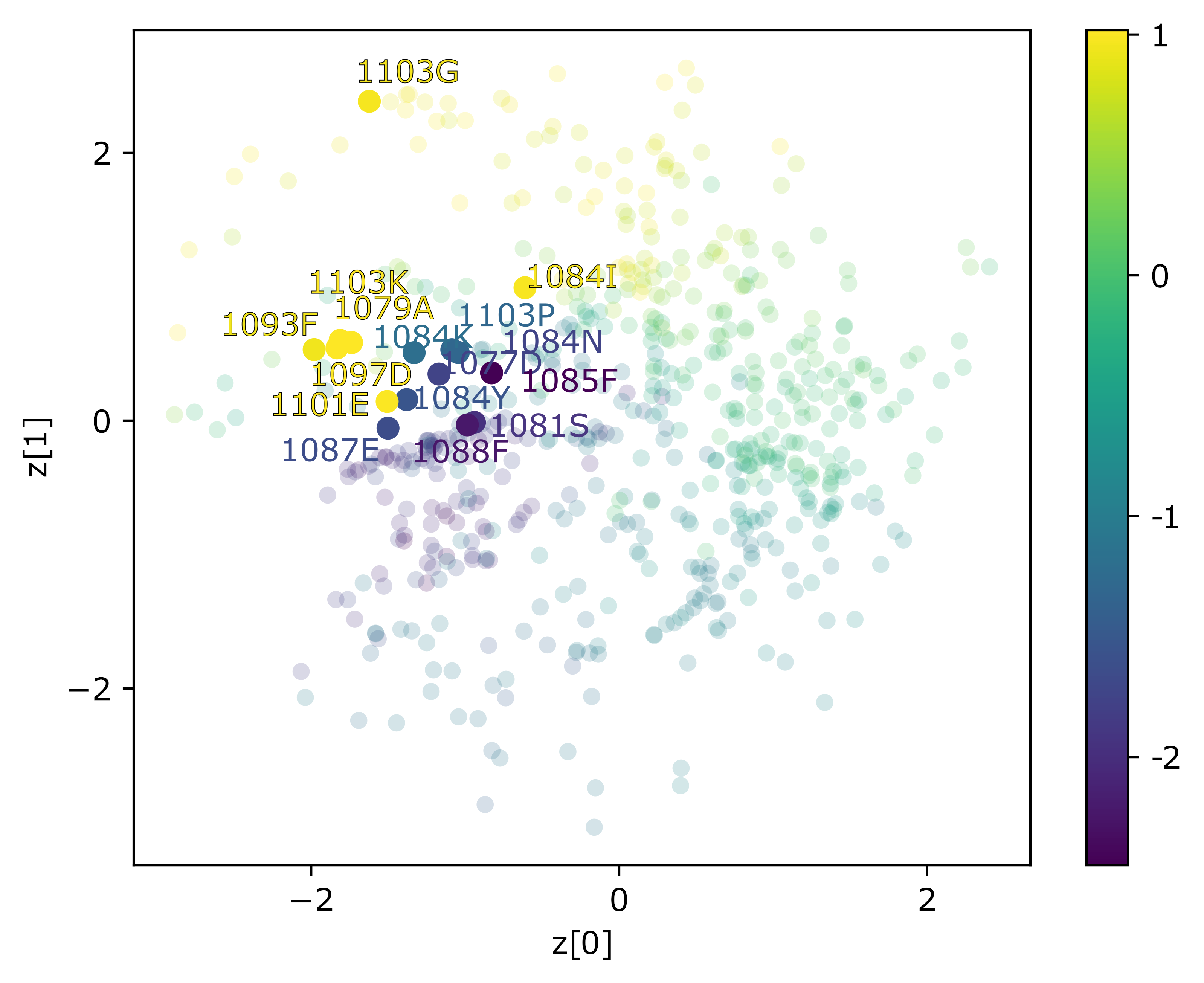

Supplement: S3 Fig — Each data point is colored according to its corresponding phenotype with transparency except the selected mutants that are colored without transparency for clarity. GOF mutants and LOF/lethal mutants at the boundary are shown. The position of E1103G in the latent space is also shown since its double mutants with the GOF mutants at the boundary cause lethality. (TIF) [file pcbi.1010999.s003.tif]

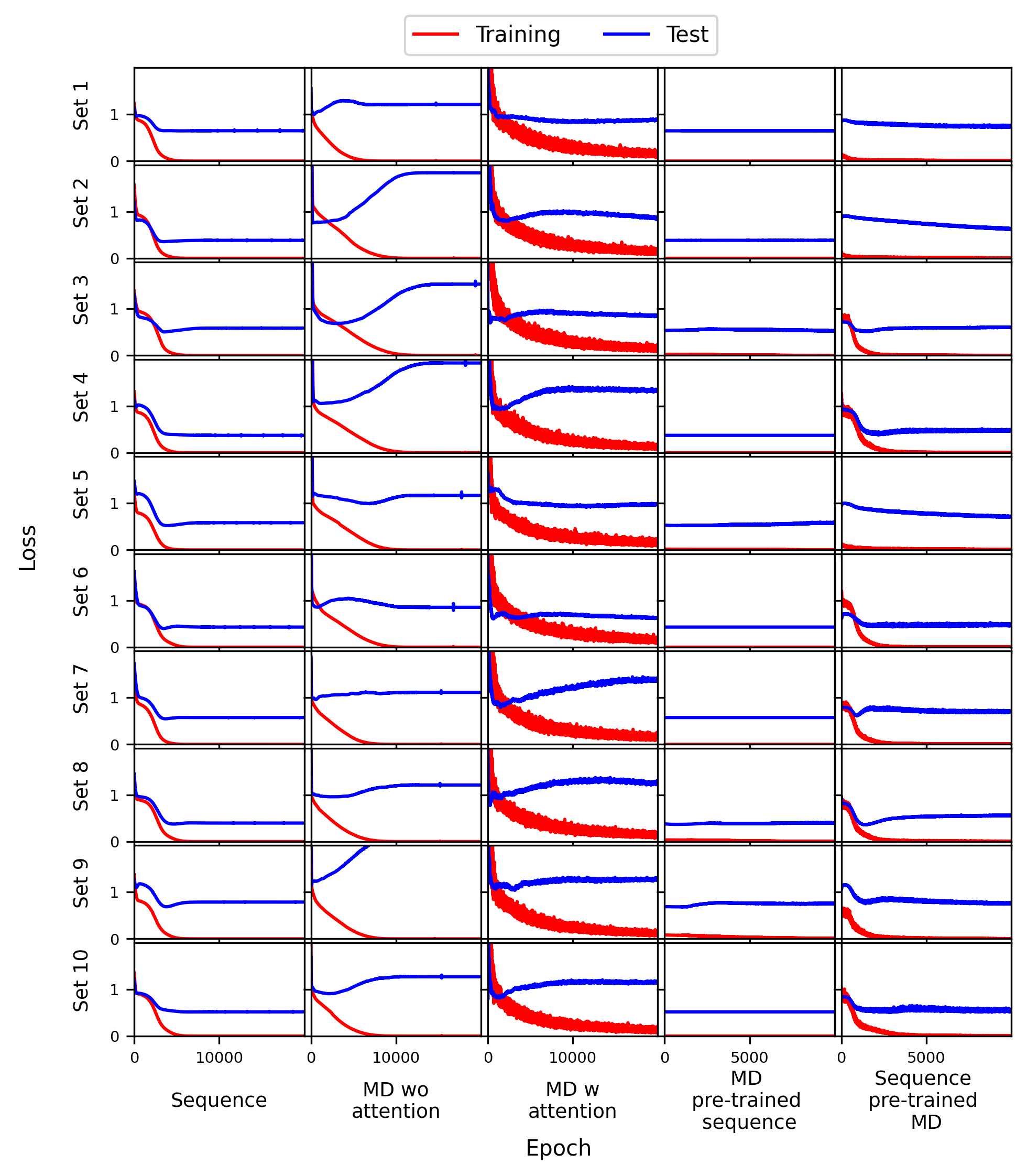

Supplement: S4 Fig — The models are trained with the input features from sequence data (Sequence), MD data without an attention layer (MD wo attention), MD data with an attention layer (MD w attention), MD and sequence data with pre-trained sequence weights (MD pre-trained sequence) and pre-trained MD weights (Sequence pre-trained MD). (TIF) [file pcbi.1010999.s004.tif]

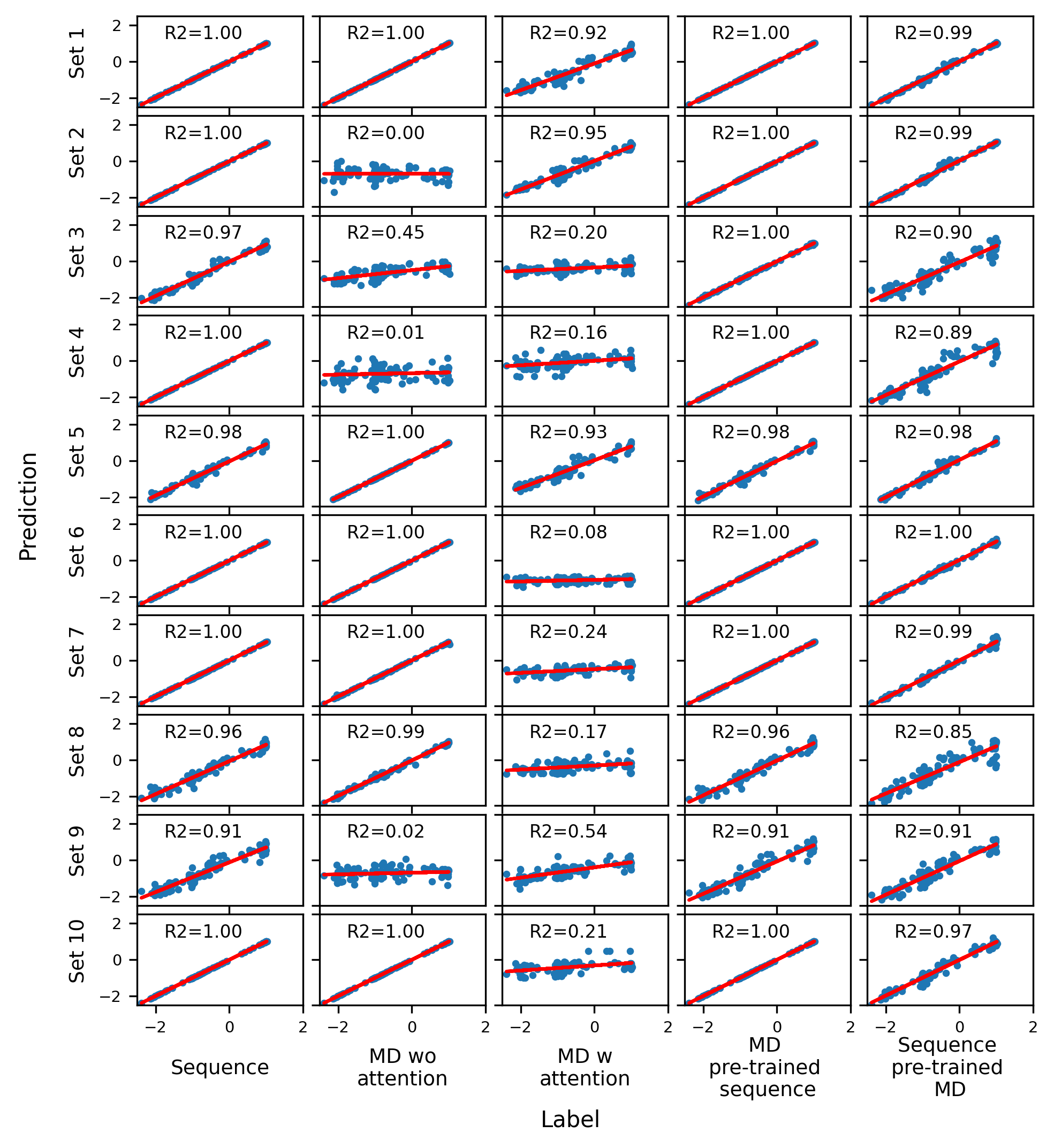

Supplement: S5 Fig — The model details are as in S2 Fig. (TIF) [file pcbi.1010999.s005.tif]

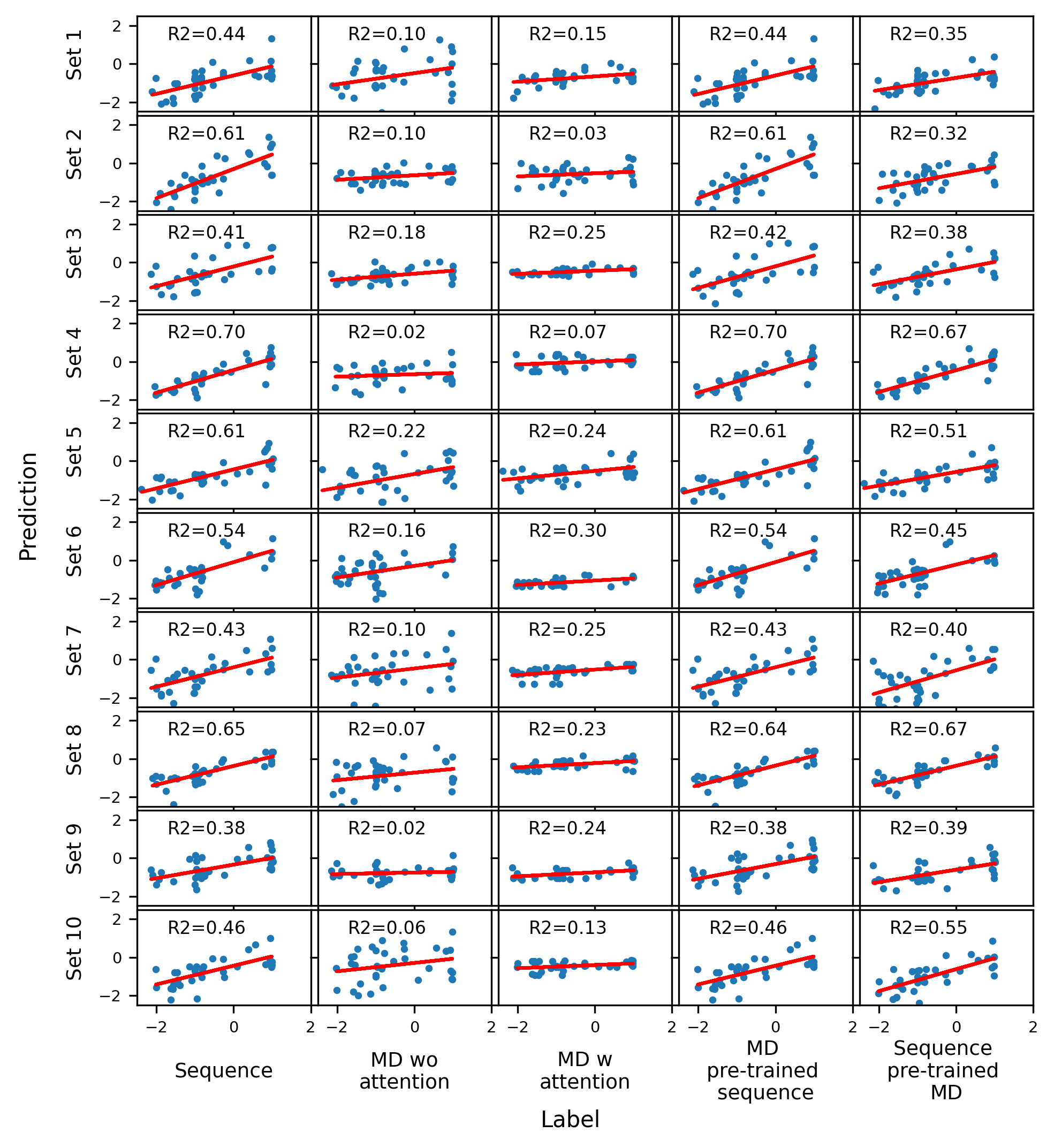

Supplement: S6 Fig — The model details are as in S2 Fig. (TIF) [file pcbi.1010999.s006.tif]

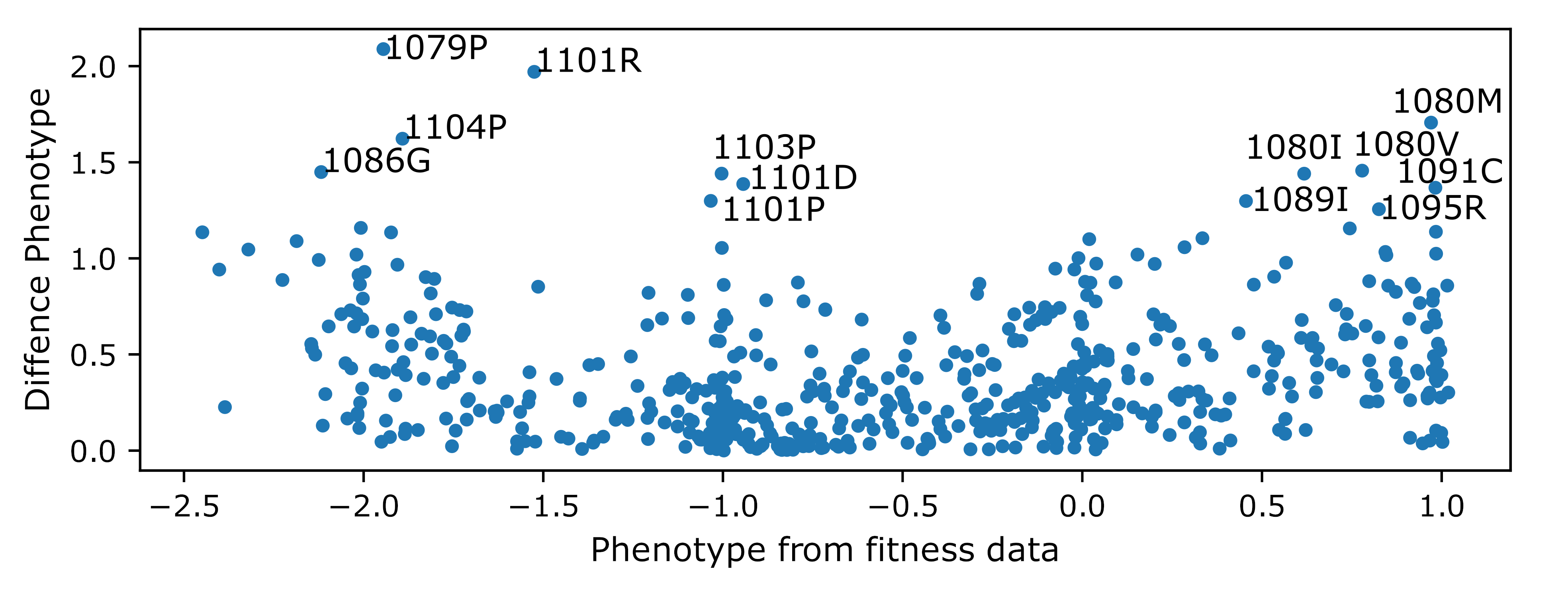

Supplement: S7 Fig — Y-axis shows the absolute value of the differences of phenotypes from the fitness and sequence data and X-axis shows the phenotypes from the fitness data. The outliers with difference larger than 1.25 are shown. (TIF) [file pcbi.1010999.s007.tif]

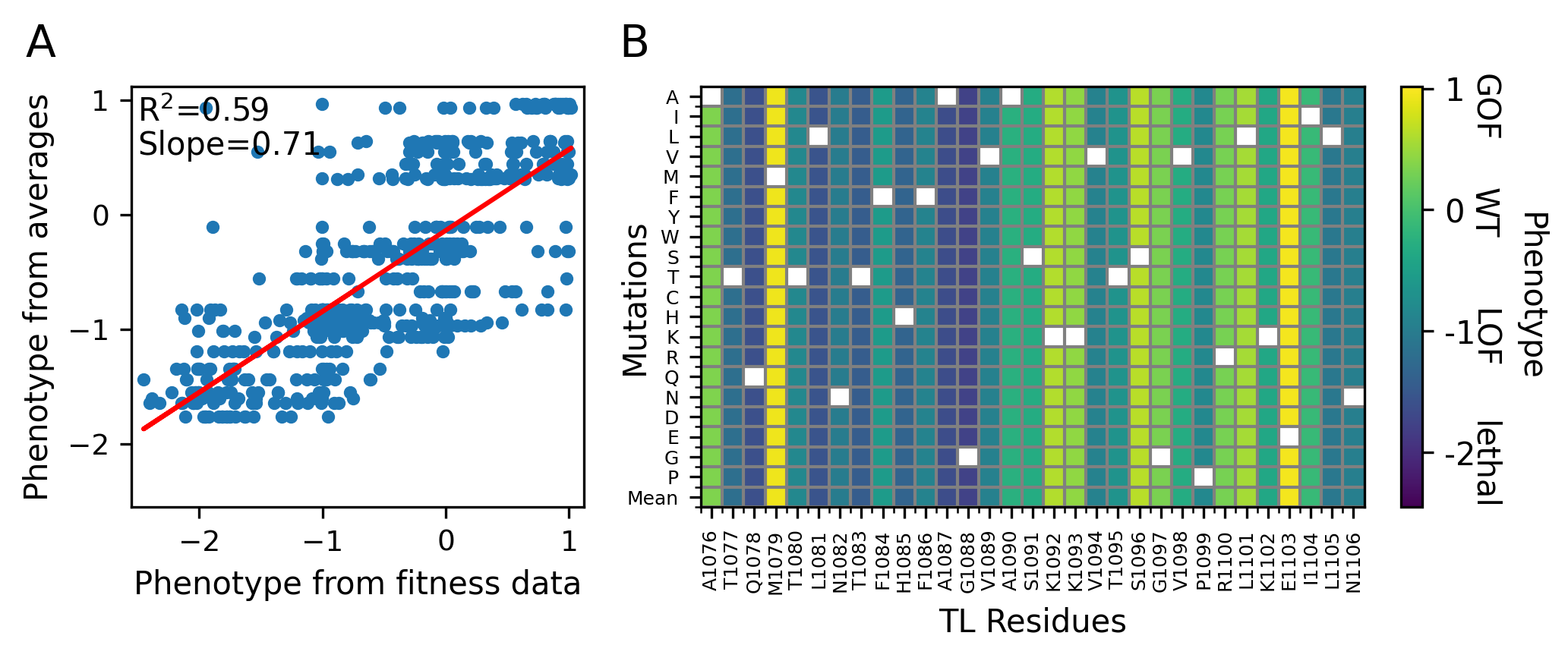

Supplement: S8 Fig — (A) the phenotypes predicted from the average values vs phenotypes from the fitness and the linear regression line (B) the average phenotypes from the training sets shown in the complete mutation map. (TIF) [file pcbi.1010999.s008.tif]

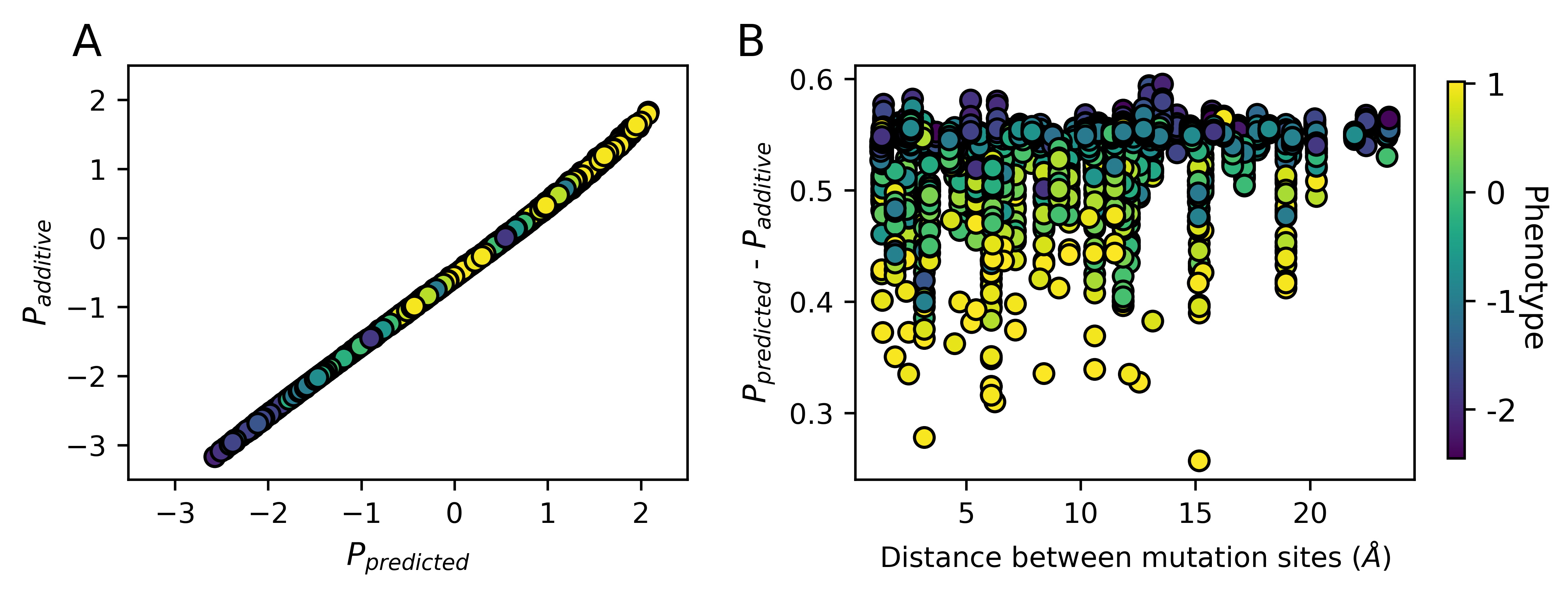

Supplement: S9 Fig — (A) Predicted phenotypes (Ppredicted) vs. additive phenotypes (Padditive) that were calculated as the sum of the predicted phenotypes of single mutants, (B) the change of difference between Ppredicted and Padditive with respect to the spatial distance between the mutation sites. Each point is colored with the single phenotype before the second mutation site (E1103G, G1097D, F1084I or Q1078S) was introduced. (TIF) [file pcbi.1010999.s009.tif]

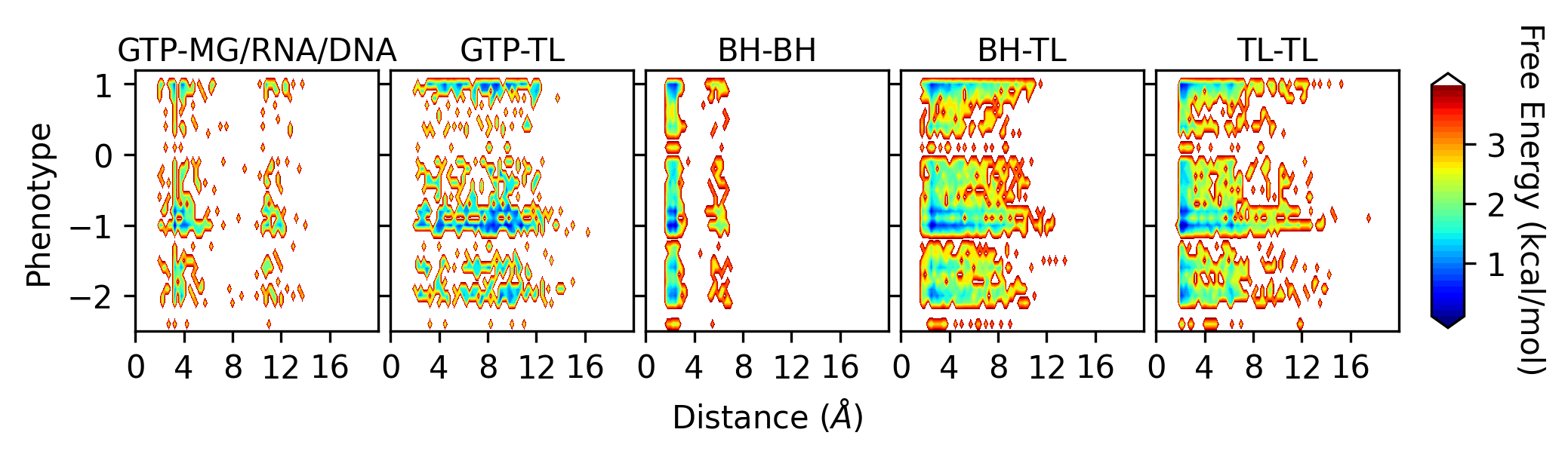

Supplement: S10 Fig — Heatmap plots of phenotypes vs average distances for the five groups of distances that are the distances between 1) GTP and MG/RNA/DNA, 2) GTP and TL residues, 3) BH and BH residues, 4) BH and TL residues, and 5) TL and TL residues for the mutants from MD simulations. (TIF) [file pcbi.1010999.s010.tif]

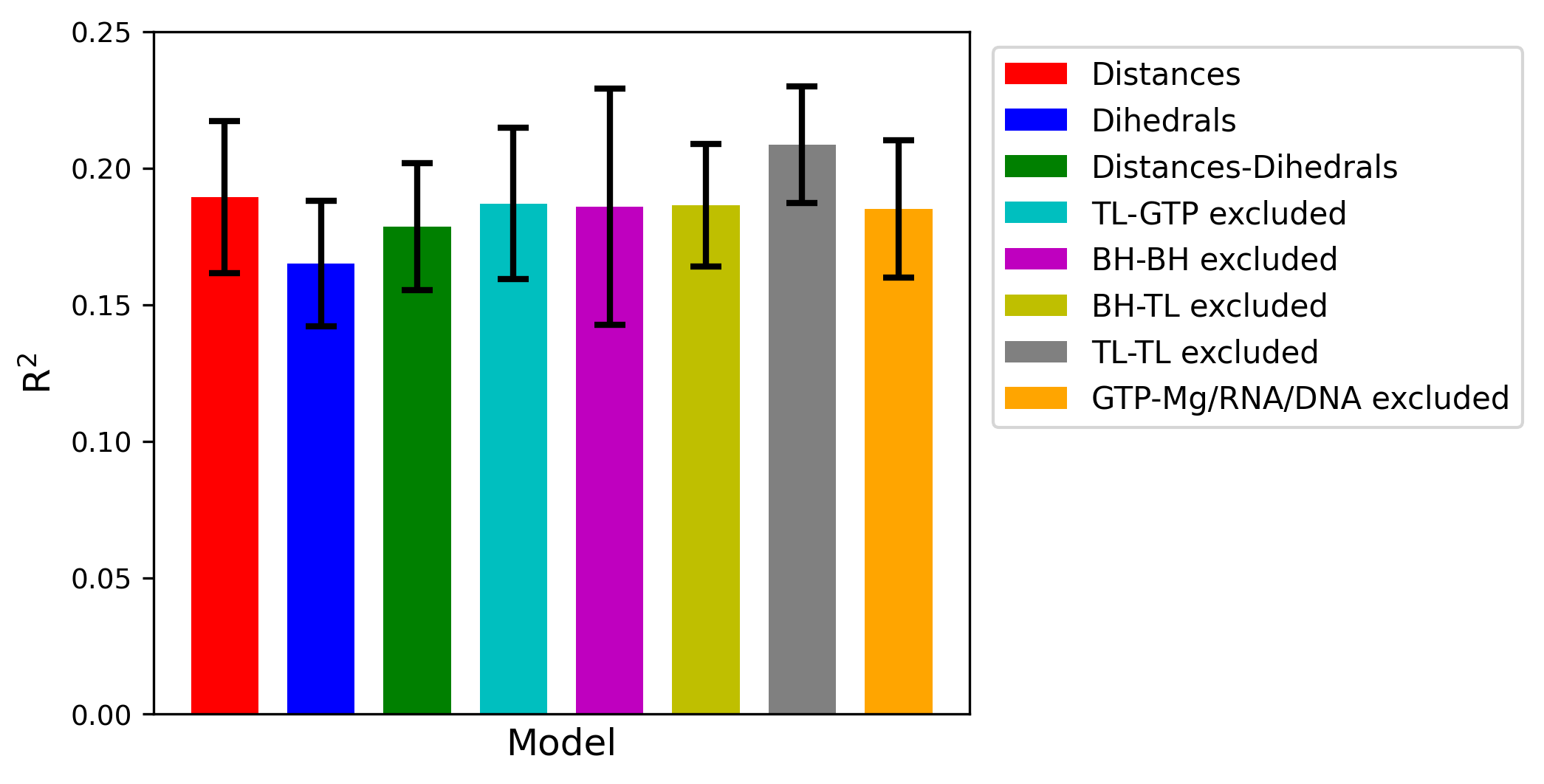

Supplement: S11 Fig — Eight model was generated using different inputs from MD data that are the five set of distances near the active site (see Fig 4), TL backbone dihedral angles, combination of distances and dihedral angles and distances by excluding one set of distance values at each model. (TIF) [file pcbi.1010999.s011.tif]

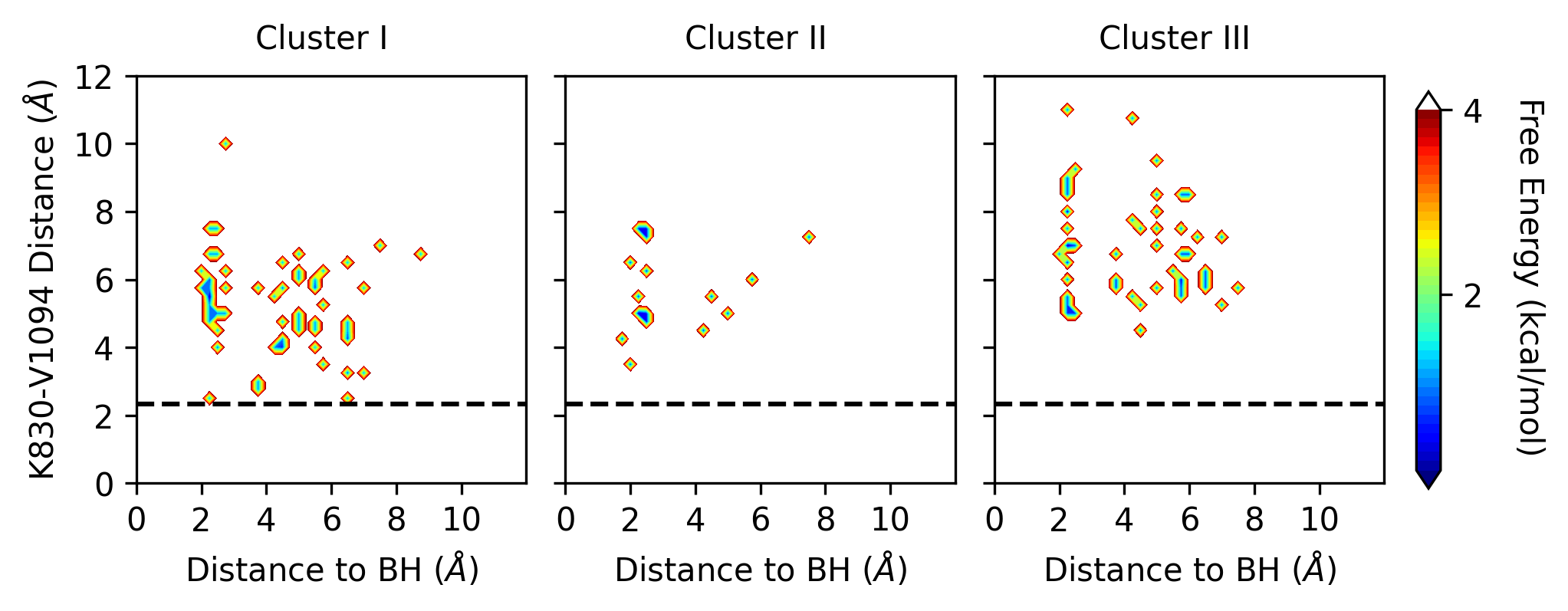

Supplement: S12 Fig — Each panel shows the plot for the members of each cluster found in the MD-VAE latent space. The dashed lines show the distance of K830 and V1094 in the WT structure. (TIF) [file pcbi.1010999.s012.tif]

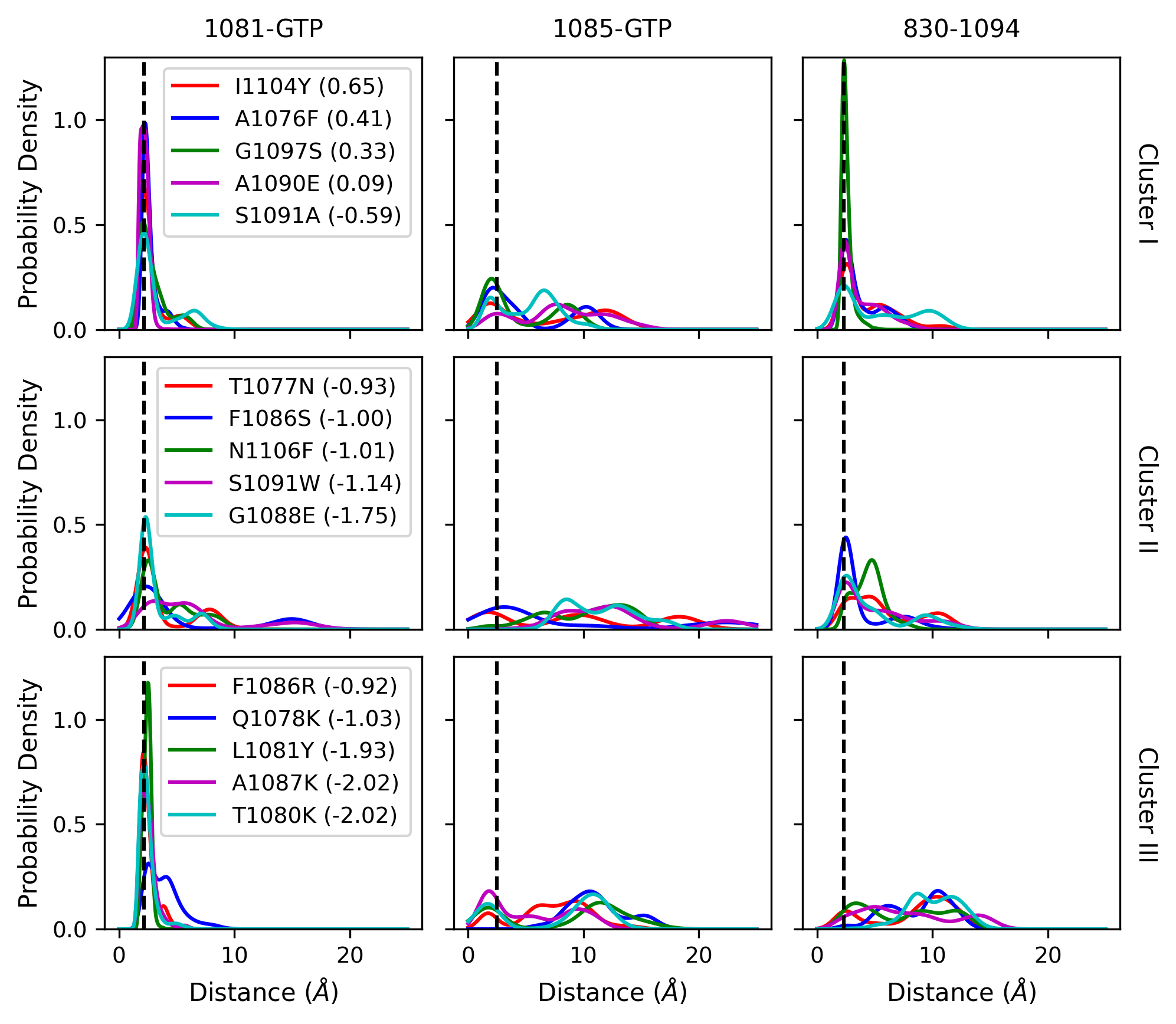

Supplement: S13 Fig — The mutants are given in the legends with their continuum phenotypes in the parentheses. (TIF) [file pcbi.1010999.s013.tif]

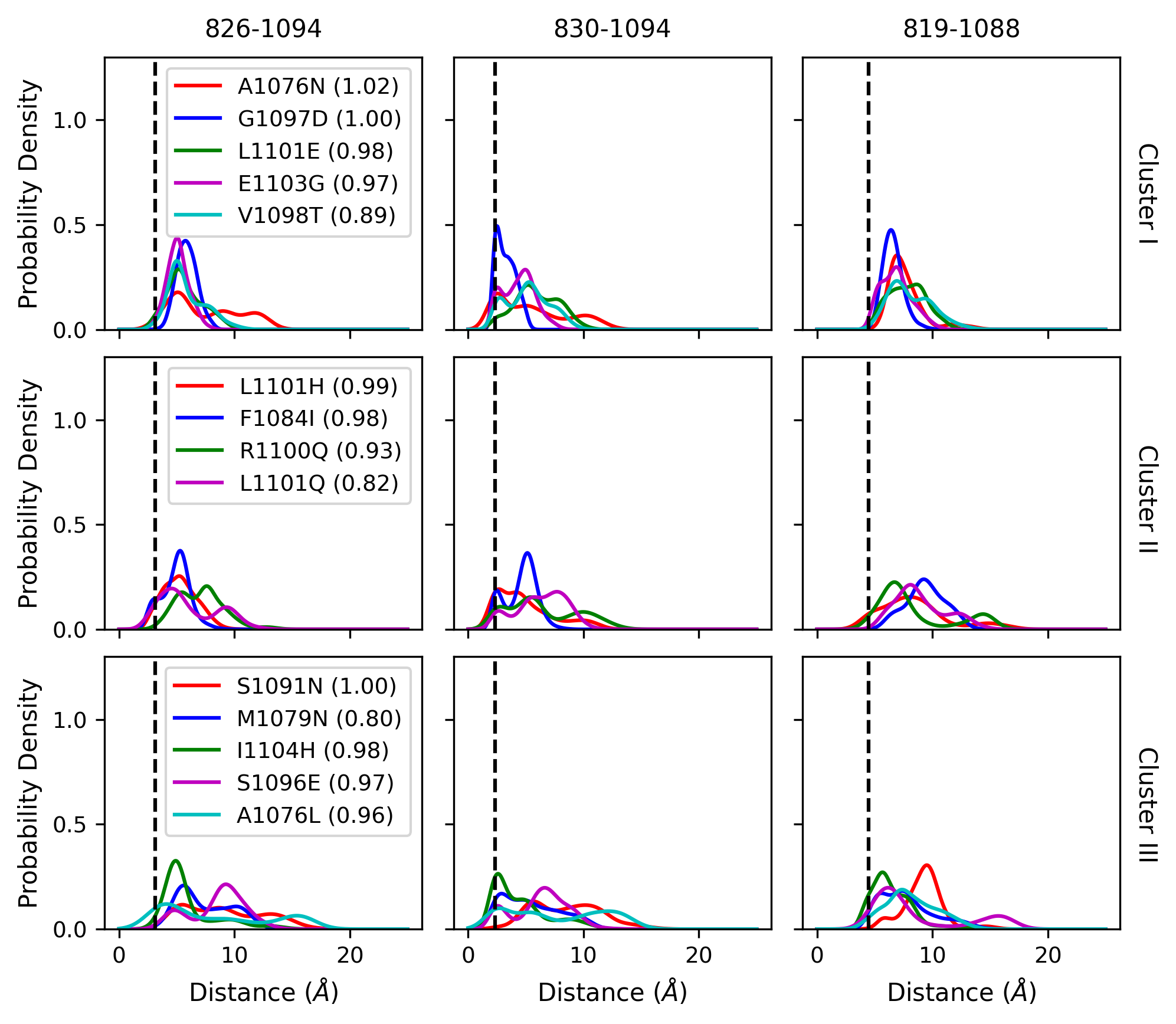

Supplement: S14 Fig — The mutants are given in the legends with their continuum phenotypes in the parentheses. (TIF) [file pcbi.1010999.s014.tif]

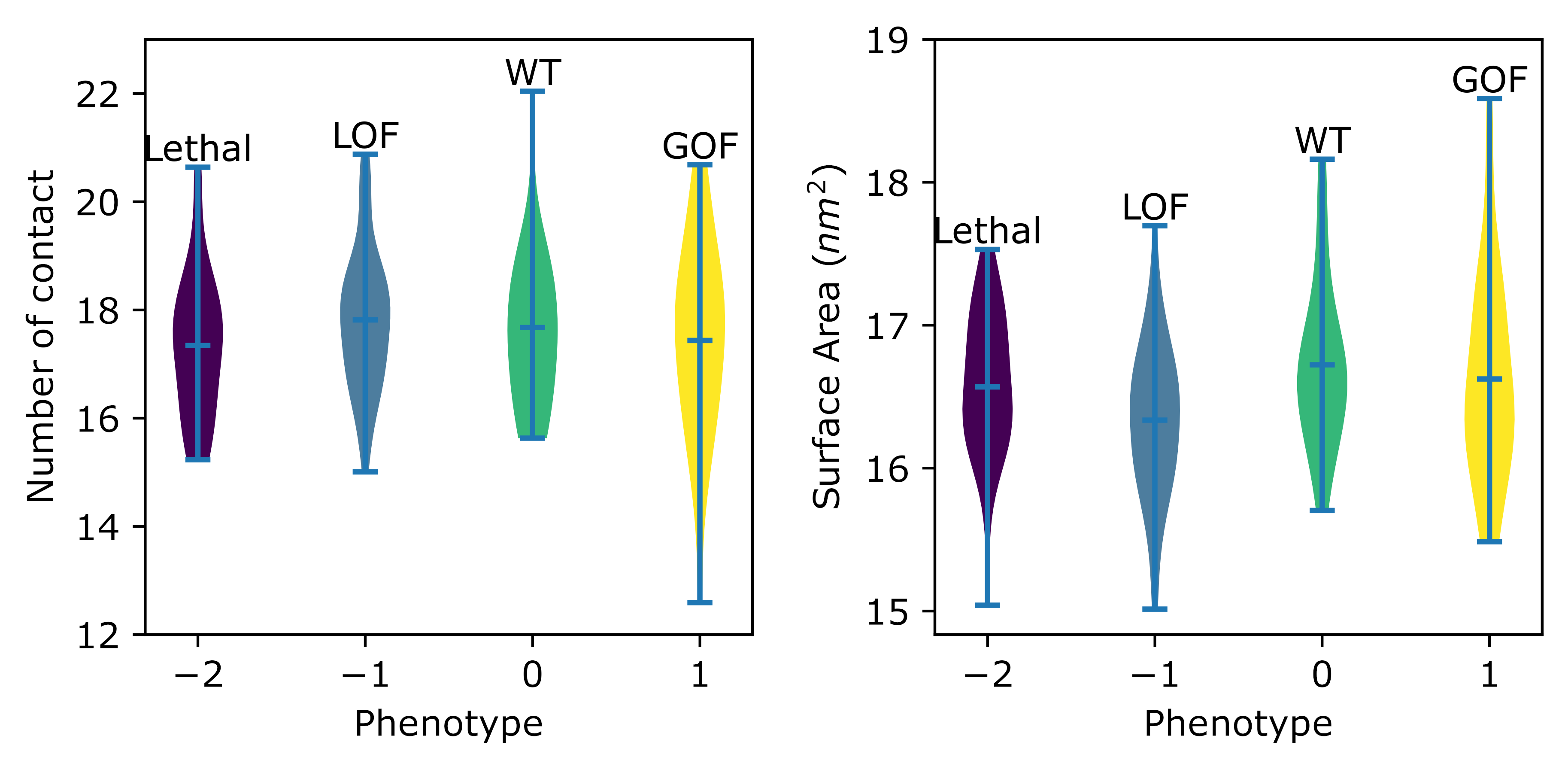

Supplement: S15 Fig — (A) Number of contacts vs. phenotypes at the hydrophobic pocket. (B) Surface area vs. phenotypes within the hydrophobic pocket. (TIF) [file pcbi.1010999.s015.tif]

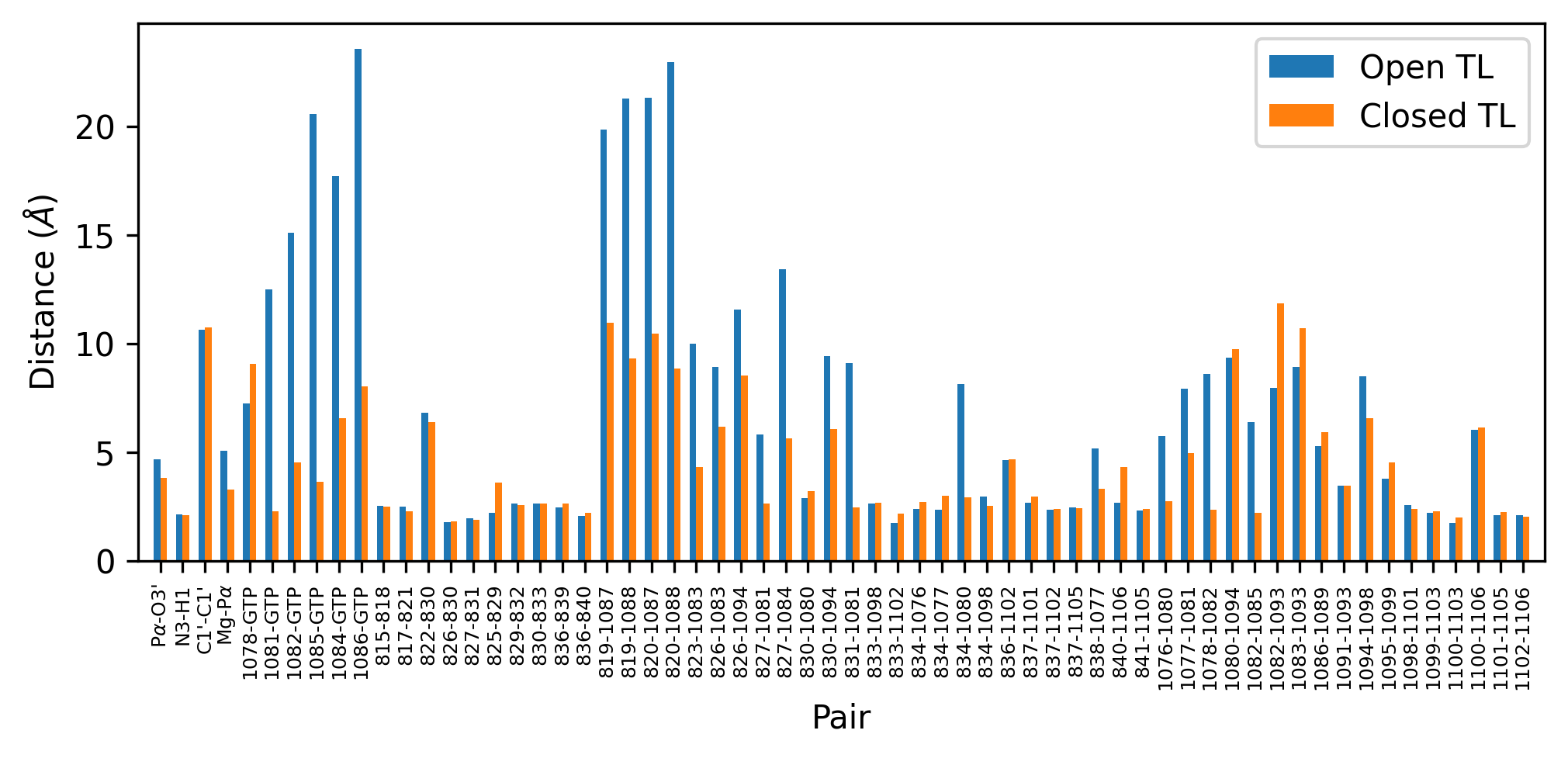

Supplement: S16 Fig — Distances were calculated by analyzing the simulations published in an earlier study. (TIF) [file pcbi.1010999.s016.tif]

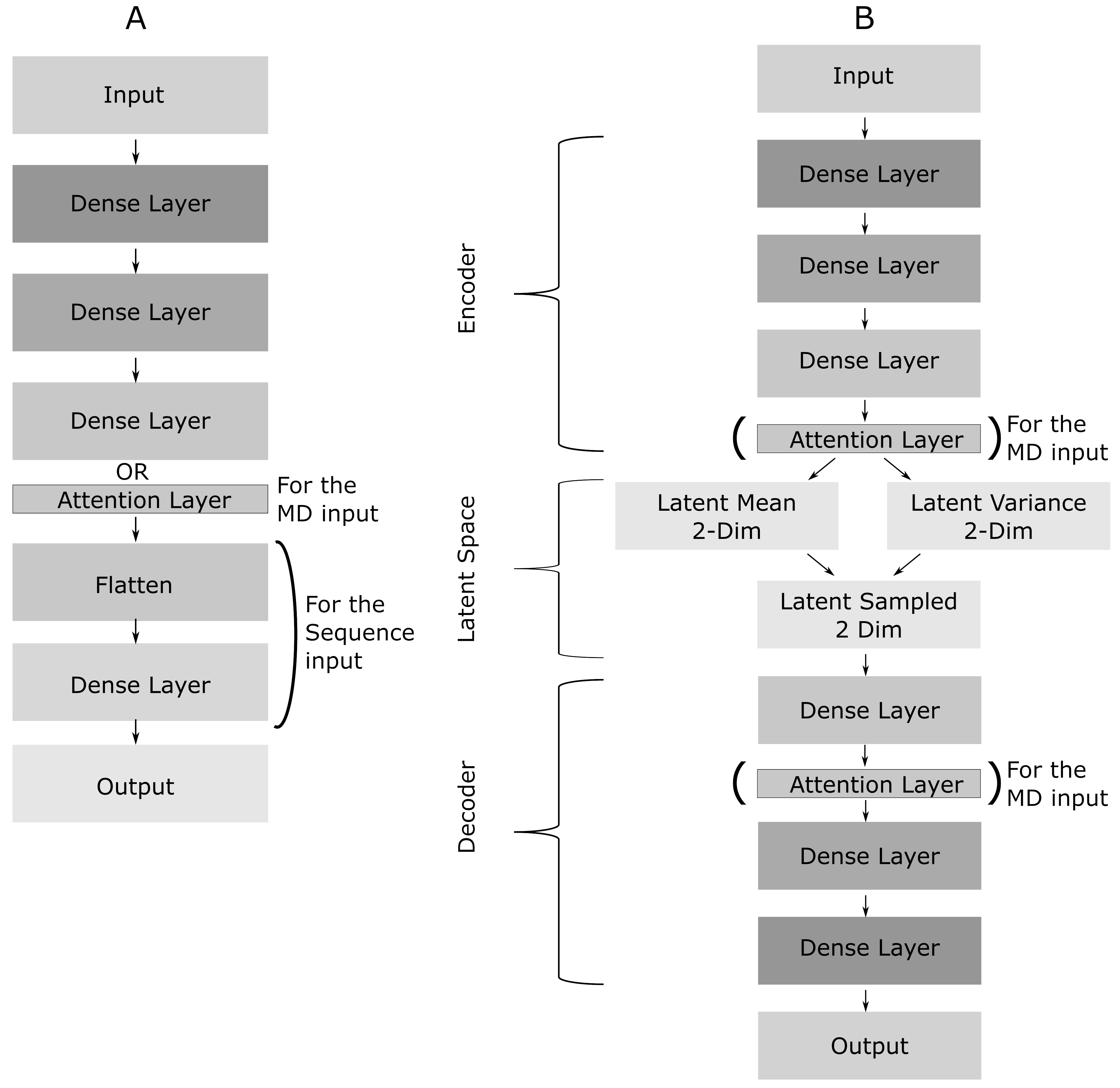

Supplement: S17 Fig — (A) The models for the prediction of continuous phenotypes have alternating layers depending on the input: For the models with fitness score as the input, three dense layers were used. For the models with the MD data as the input, three dense layers or two dense layers and one attention layer were used. For the models with amino acid sequence as the input, two-dimensional matrix at the third dense layer was flattened out and passed through another dense layer. (B) VAE model was applied to the fitness scores as three dense layers on the encoder and decoder models. It was applied to the MD data with additional attention layers on the encoder and decoder. (TIF) [file pcbi.1010999.s017.tif]

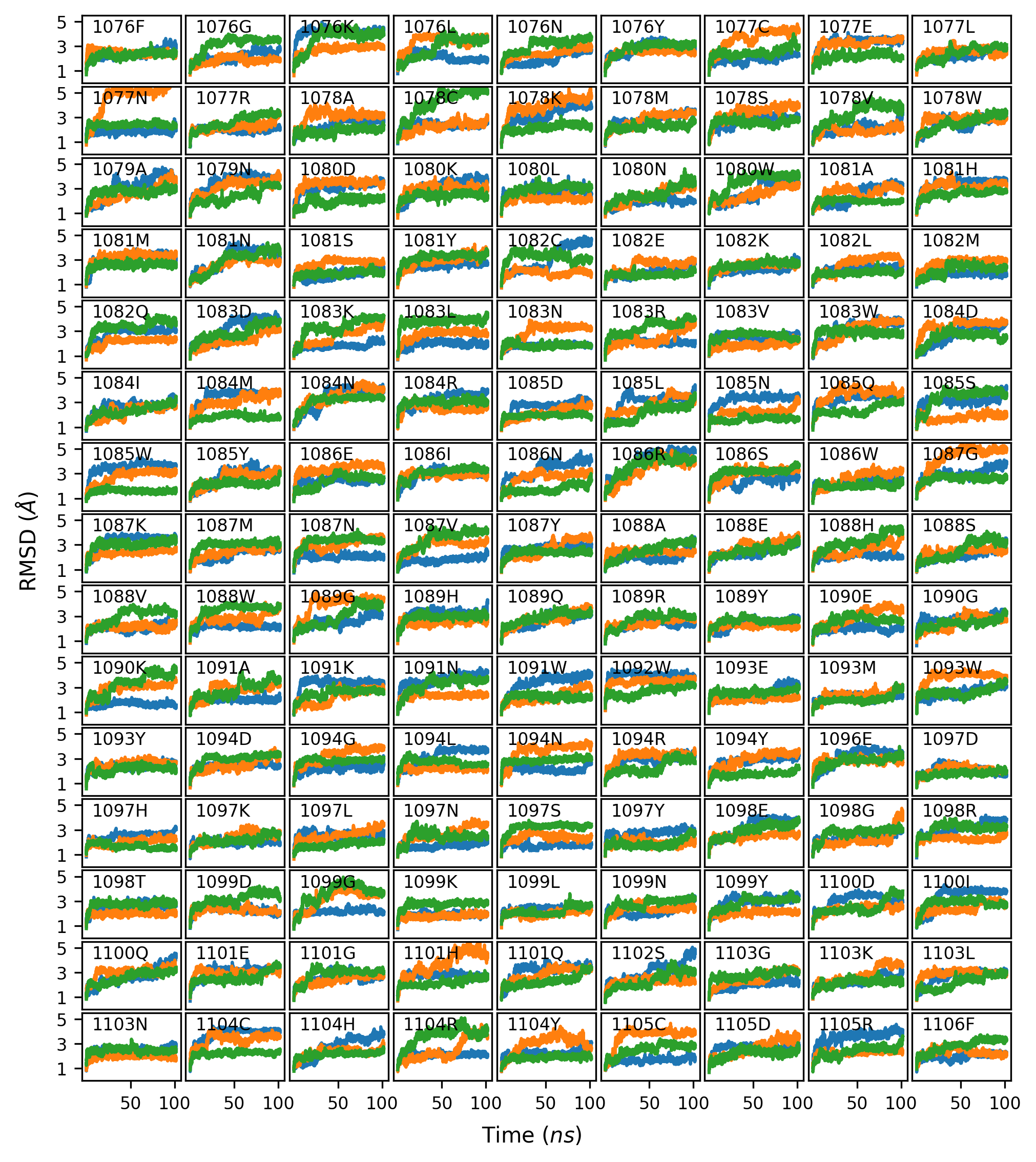

Supplement: S18 Fig — RMSD values from three replicate simulations were represented with different colors. There are not large changes in RMSD for TL suggesting that TL is retaining its overall conformation for the mutants within the simulation time scale. (TIF) [file pcbi.1010999.s018.tif]

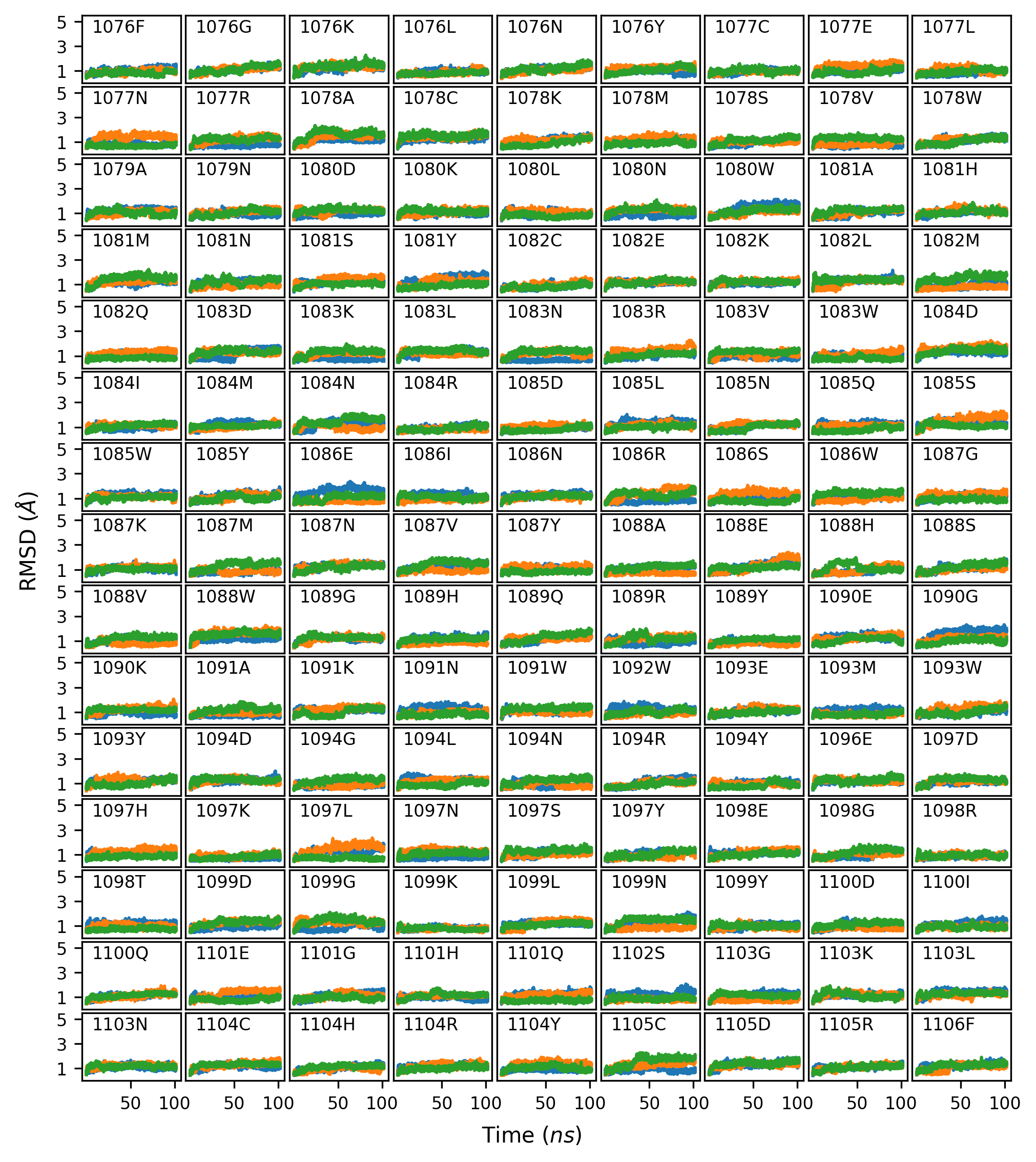

Supplement: S19 Fig — RMSD values from three replicate simulations were represented with different colors. There are not large changes in RMSD for BH suggesting that BH is retaining its overall conformation for the mutants within the simulation time scale. (TIF) [file pcbi.1010999.s019.tif]

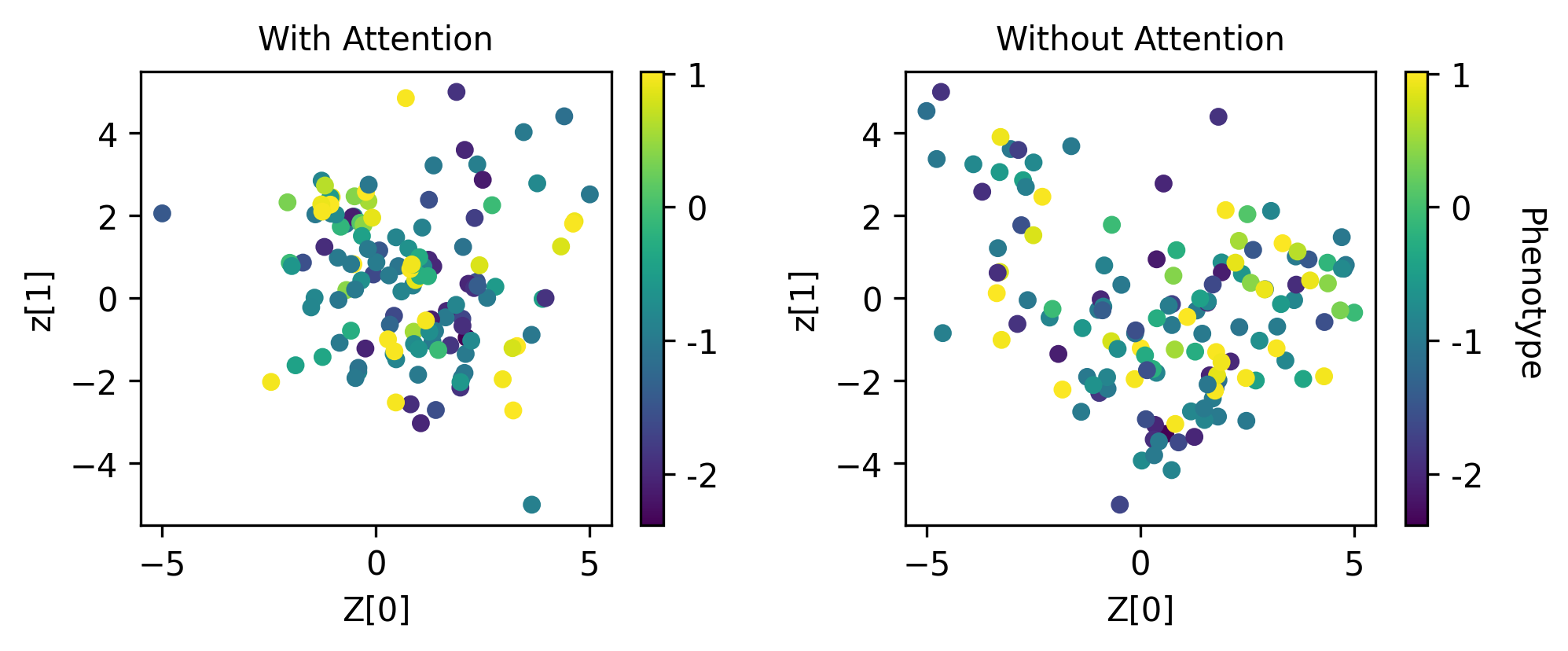

Supplement: S20 Fig — (TIF) [file pcbi.1010999.s020.tif]

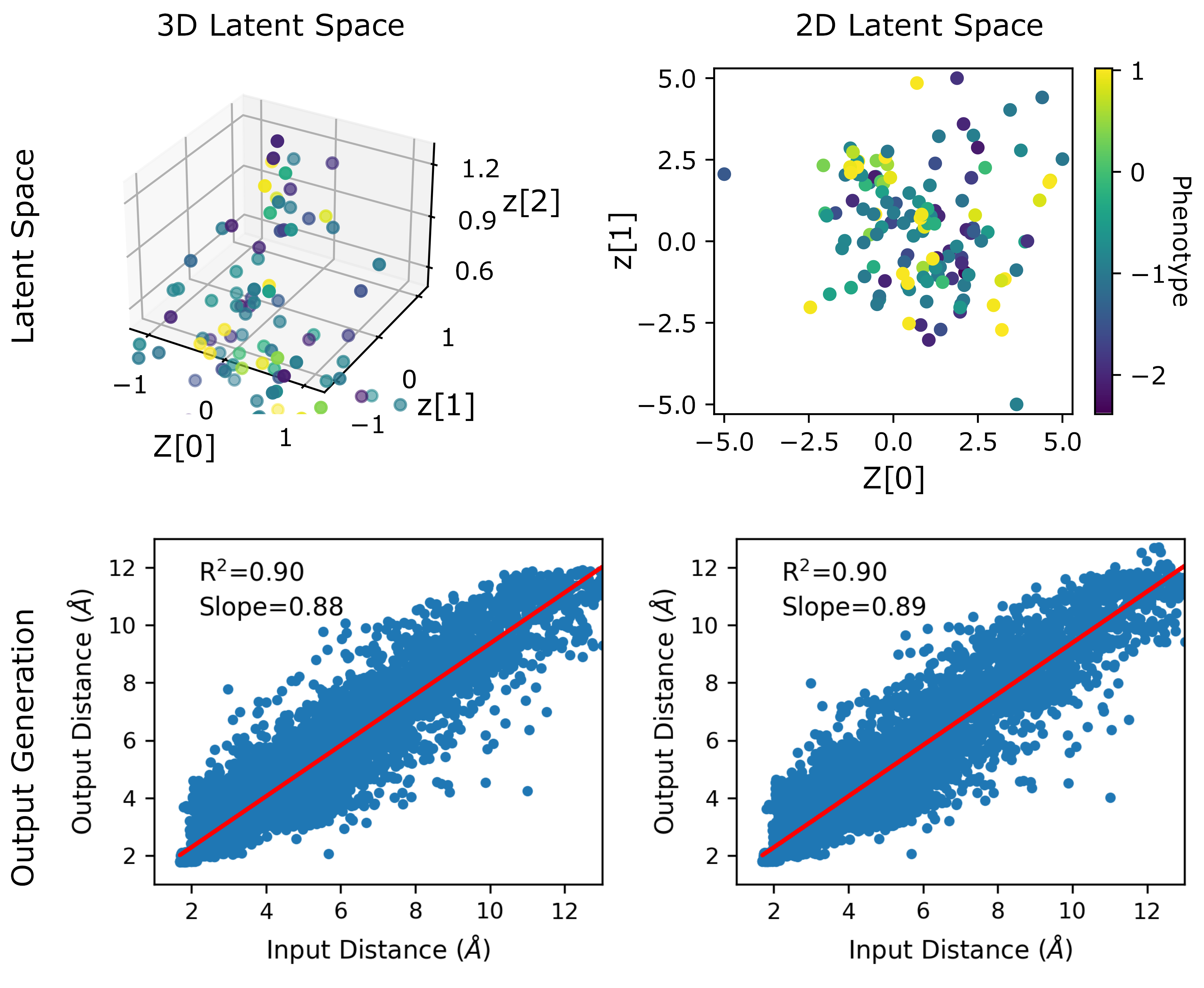

Supplement: S21 Fig — The distribution of mutants on the latent spaces (top) and generative performances (bottom) of the VAE models with 3D (left) and 2D (right) latent spaces using MD data as the input. (TIF) [file pcbi.1010999.s021.tif]

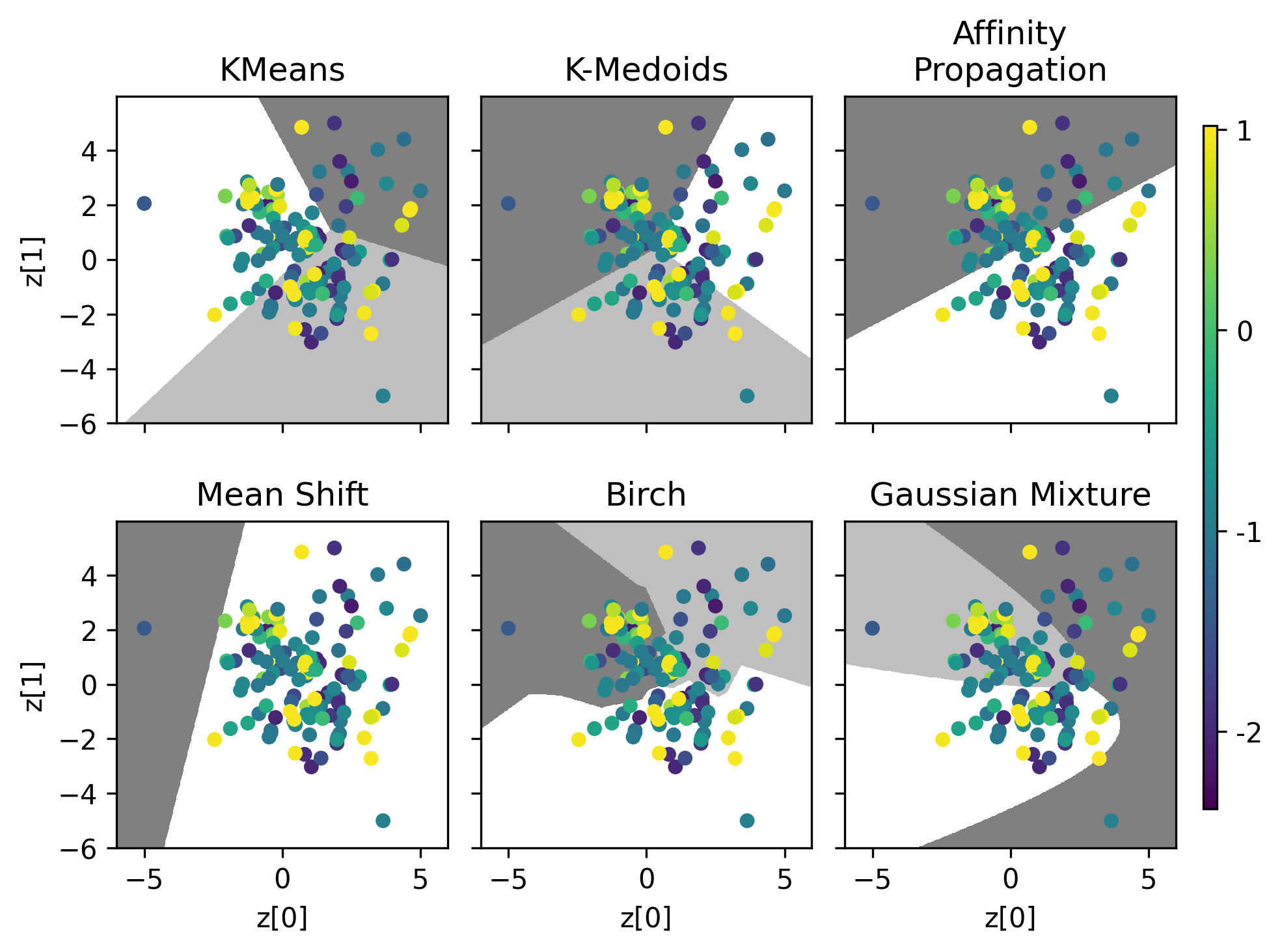

Supplement: S22 Fig — Each cluster is shown in colors from white to different shades of grey; mutants are scattered, and color coded with corresponding phenotypes. (TIF) [file pcbi.1010999.s022.tif]

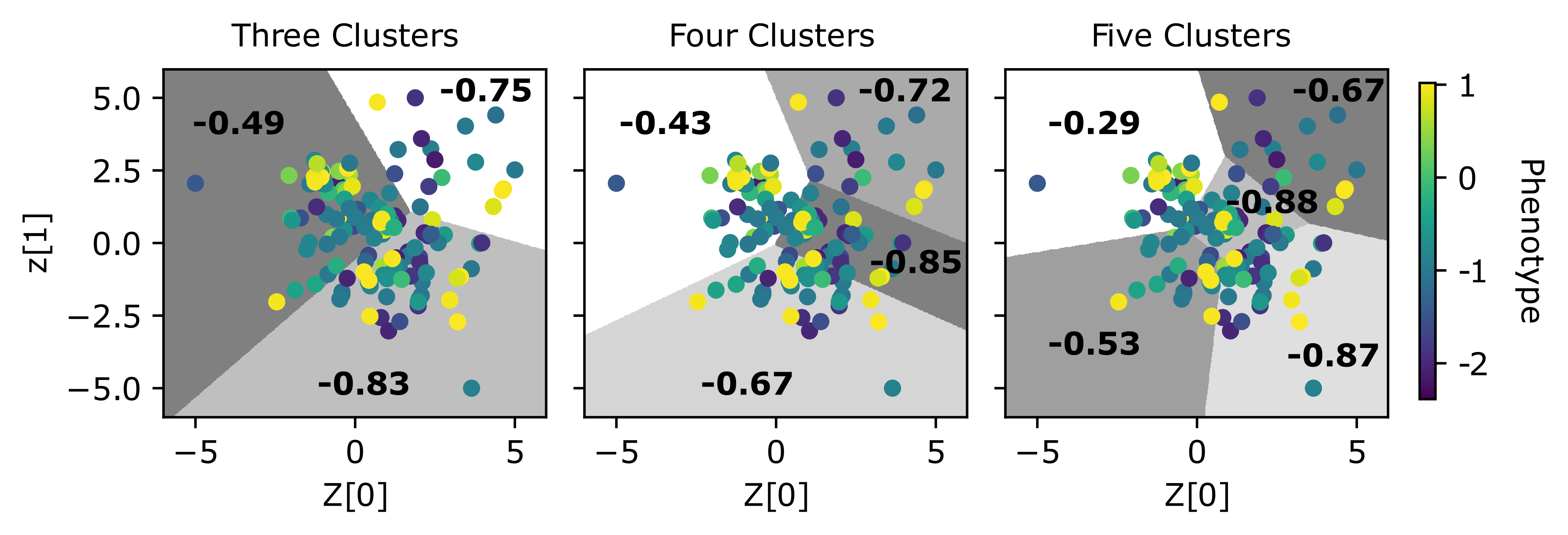

Supplement: S23 Fig — Each cluster is shown in colors from white to different shades of grey; mutants are scattered, and color coded with corresponding phenotypes; at each cluster the average phenotypes of the mutants are shown. (TIF) [file pcbi.1010999.s023.tif]

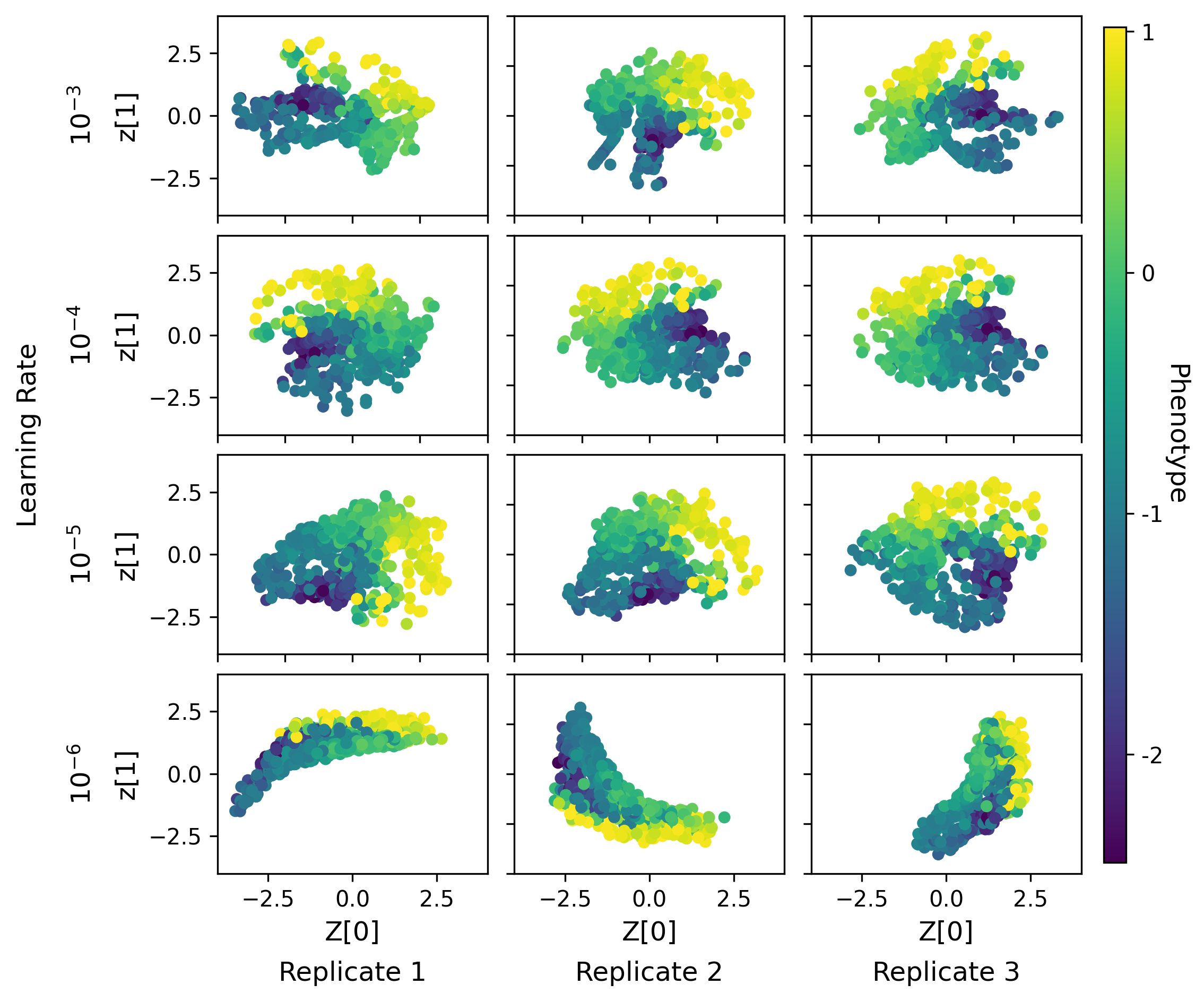

Supplement: S24 Fig — Models with 10−6 learning rate were not converged. Models with 10−3 learning rate tend to be stuck in a local minimum loss. The models with 10−4 and 10−5 learning rates provided similar latent spaces without any convergence problem, therefore a learning rate of 10−4 was used for fitness-based models. (TIF) [file pcbi.1010999.s024.tif]

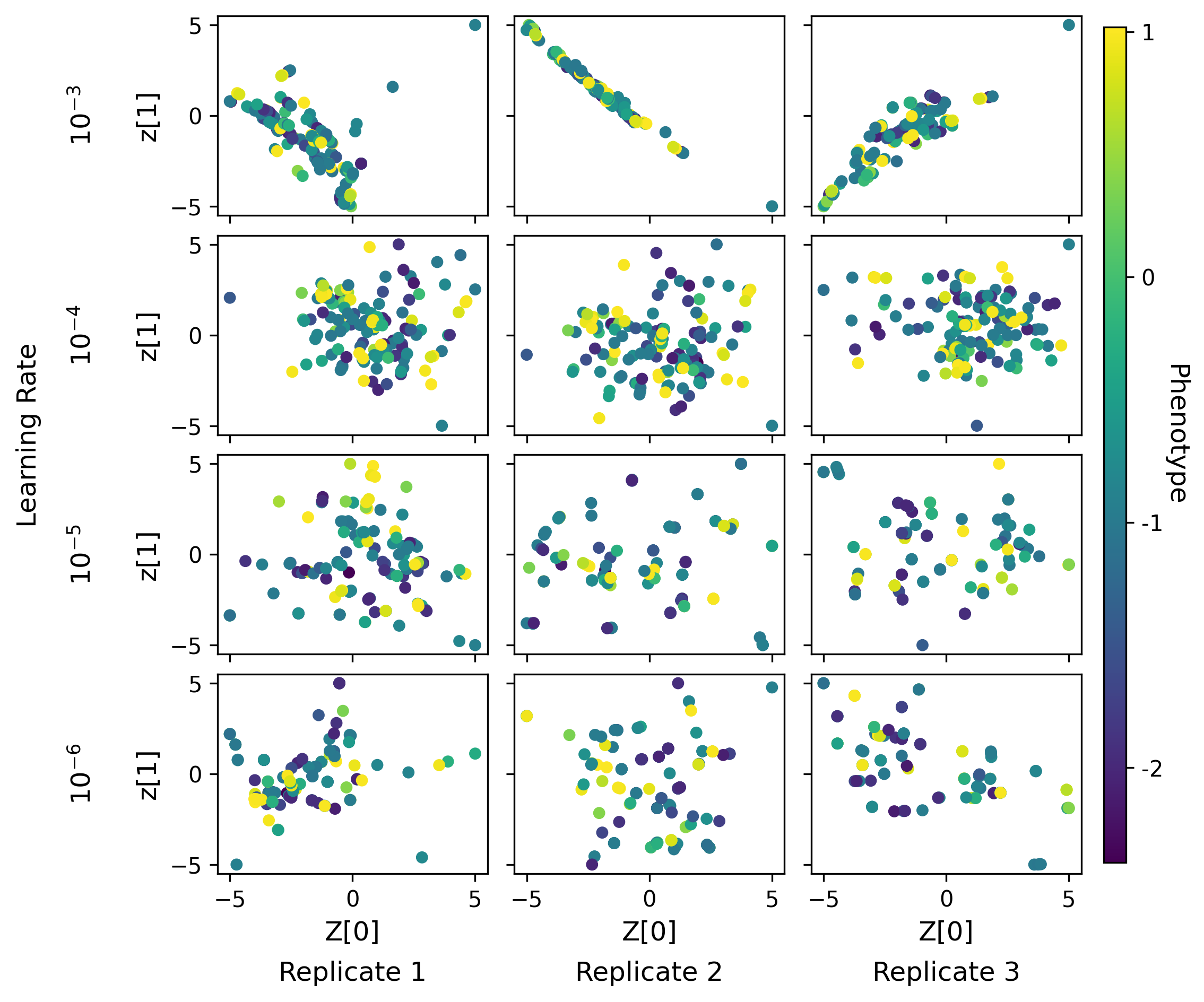

Supplement: S25 Fig — The same trend with the S20 Fig is observed. Learning rate of 10−4 provided the most visual separation of phenotypes, therefore it was used for the MD data models. (TIF) [file pcbi.1010999.s025.tif]

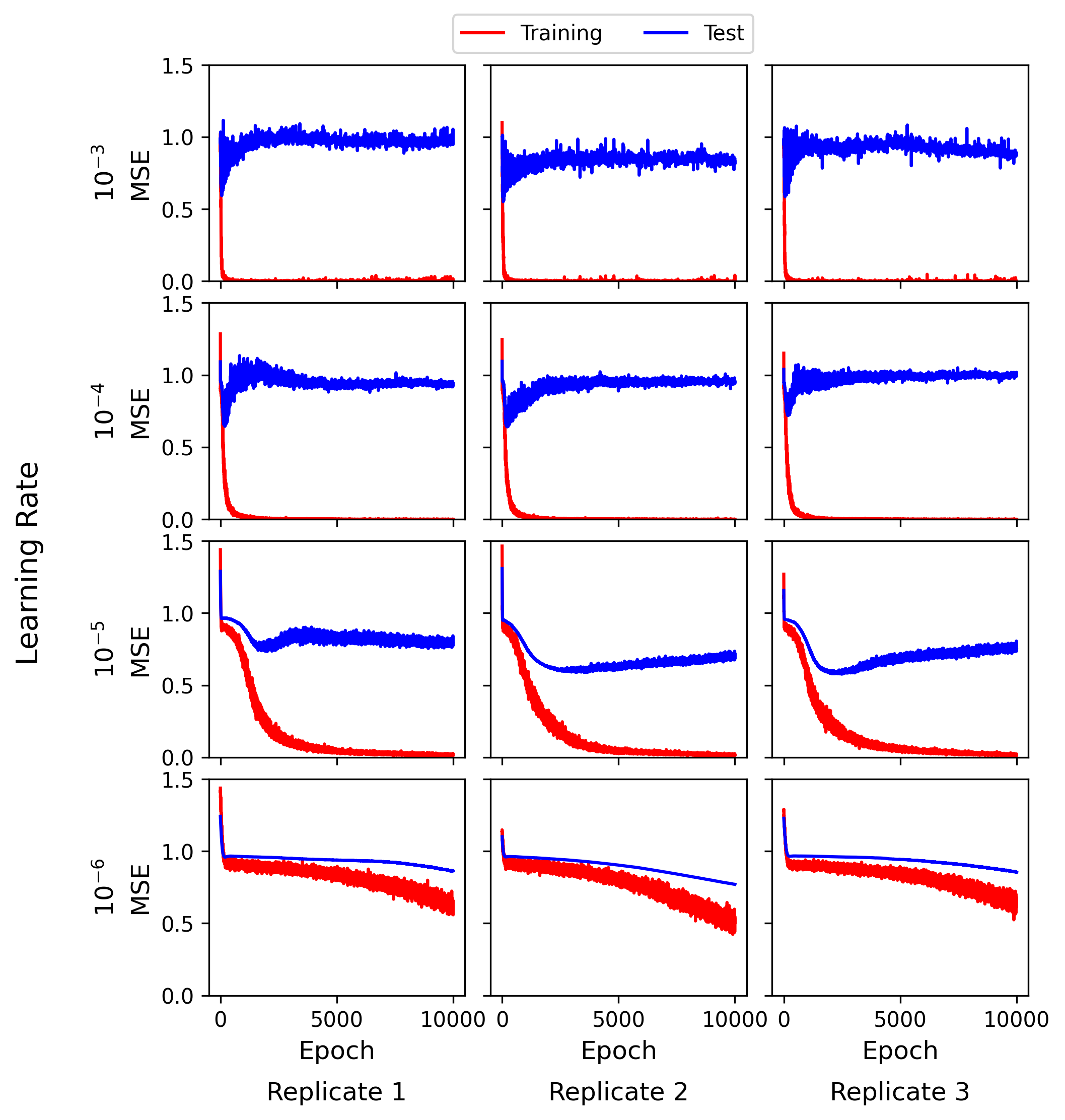

Supplement: S26 Fig — Learning rate of 10−5 provided the minimum losses for the test sets, therefore it was used for the sequence models. (TIF) [file pcbi.1010999.s026.tif]
