# Supplementary material for: Characterization of RNA polymerase II trigger loop mutations using molecular dynamics simulations and machine learning
Source: PLoS Comput Biol. 2023 Mar 22;19(3):e1010999. doi: 10.1371/journal.pcbi.1010999 (PMC10069792; doi:10.1371/journal.pcbi.1010999)
Supplement: S1 Table — (DOCX) [file pcbi.1010999.s027.docx]

**Table S1.** The summary of deep learning models

| Model | Optimizer | Learning Rate | Epochs | Batch Number | Loss Function |
| --- | --- | --- | --- | --- | --- |
| Fitness – Prediction | Adam | 10^-4^ | 2000 | 4 | MSE |
| Fitness – VAE | Adam | 10^-4^ | 5000 | 4 | MSE+KL |
| Sequence – Prediction | Adam | 10^-5^ | 20000 | 100 | MSE+KL |
| MD – Prediction | Adam | 10^-5^ | 20000 | 100 | MSE+KL |
| MD – Prediction with Attention | Adam | 10^-5^ | 20000 | 4 | MSE |
| MD – VAE | Adam | 10^-4^ | 5000 | 4 | MSE+KL |
