# Supplementary material for: Characterization of RNA polymerase II trigger loop mutations using molecular dynamics simulations and machine learning
Source: PLoS Comput Biol. 2023 Mar 22;19(3):e1010999. doi: 10.1371/journal.pcbi.1010999 (PMC10069792; doi:10.1371/journal.pcbi.1010999)
Supplement: S2 Table — T-test was performed by assuming the two populations have different variance. Clusters I, II and III corresponds to the clusters shown in Fig 6A. (DOCX) [file pcbi.1010999.s028.docx]

**Table S2.** Statistical analysis of the phenotypes in the clusters obtained from VAE model using MD data. T-test was performed by assuming the two populations have different variance. Clusters I, II and III corresponds to the clusters shown in Figure 6A

| Metric | I | II | III | I-II | I-III | II-III |
| --- | --- | --- | --- | --- | --- | --- |
| Mean | -0.49 | -0.75 | -0.83 |  |  |  |
| Standard error | 0.11 | 0.26 | 0.14 |  |  |  |
| T-statistics |  |  |  | 0.90 | 1.96 | 0.29 |
| P-value |  |  |  | 0.378 | 0.053 | 0.771 |
