# Supplementary material for: Characterization of RNA polymerase II trigger loop mutations using molecular dynamics simulations and machine learning
Source: PLoS Comput Biol. 2023 Mar 22;19(3):e1010999. doi: 10.1371/journal.pcbi.1010999 (PMC10069792; doi:10.1371/journal.pcbi.1010999)
Supplement: Attachment — Submitted filename: response_reviewer_comments.pdf [file pcbi.1010999.s031.pdf]

RESPONSE TO REVIEWER COMMENTS

We thank the reviewers for their insightful comments. We revised the manuscript accordingly and we are providing a point-by-point response below.

Comments to the Authors:
Please note here if the review is uploaded as an attachment.

Reviewer #1: In this work, the authors have combined MD simulations and ML techniques to characterize the relation between the trigger loop mutants and the phenotypes. They have found out that the TL sequences could serve as the appropriate variables to predict the phenotypes while inclusion of the structural information extracted from the MD simulations would not further improve the prediction. Moreover, they have applied the variational auto-encoder model to correlate the GOF, LOF or lethal mutations with the key distances, such as the TL-BH distance, the TL-GTP distance, etc, which could provide the structural insights into the influence of the mutations on the phenotypes. Overall, this work is interesting and has applied the ML techniques to the critical enzyme Pol II to make prediction about the TL mutations, which could advance our knowledge about the role of TL in transcription. I think it fits the scope of Plos Comput. Bio.. However, I have several comments for the authors to address.

Major comments:
1. In the introduction, please review how the machine learning (ML) frameworks have been applied to predict the phenotypes upon mutations in other biological systems or justify why you decide to choose ML in the current work.

Machine learning is increasingly being used to describe the effects of mutations in biological systems. The advantage of ML is that it can handle complex relationships derived from high-dimensional data. We have added a brief literature review of ML applications on mutation effects on protein function into the introduction section of the manuscript (see page 5-6).

2. Page 7, line 142: It is not clear if the training set has included the mutations on all the residues on the TL. This information should be provided and the complete training set (including the mutation and the phenotype) could be added in the SI.

The training set consists of 83 mutants with known phenotypes. In the Supplementary Spreadsheet 1, we provided the mutations, fitness data, known and predicted phenotypes. We also added a sub-sheet that has only the training set. We clarified the given data in the results section (see page 9).

3. Page 8, line 147: "… the fact that these TL residues are critical for function." Please specify how they are critical for what function.

We have added a more detailed explanation of the proposed functions of these residues (see page 9).

4. Page 8, line150-157: In this part, the authors have discussed the mutations on several amino acids, such as A1076, M1079, G1097, L1101, K1092 and K1093, would destabilize the open TL state and result in GOF, as it would facilitate the closure of the TL. Can the authors perform MD simulations for some mutations to validate their proposal?

These residues were hypothesized by other studies to stabilize the open TL through formation of a hydrophobic pocket (Barnes et al., 2015, Mol. Cell 59, 258–269; Qiu C et al., 2016, PLoS Genet, 12(11): e1006321) and the mutations on these residues cause GOF function supposably by disrupting this hydrophobic pocket. The present study is based on mutant simulations of these residues, but with a closed TL. From these simulations, we show that the hydrophobic pocket is partly disrupted for selected mutants (Fig. 6F-H) when the TL is closed. Additional simulations of these mutants in just the open state would be less informative since the TL is more flexible and the hydrophobic pocket is not formed in that case. What would be needed to fully investigate the proposed involvement of the hydrophobic pocket are simulations that cover the TL closing transition. We have published such work previously for the wild-type sequences (Wang et al., 2013, Biophys. J, 105, 765-775), but it remains computationally very expensive to repeat such work for a number of mutations. Such efforts are outside of the scope of the present study, but we agree that these would be excellent topics for follow-up work in future studies.

5. Page 9: how to define the “transition”? Can they provide some examples for the mutations that lead to the direct transition from lethal to GOF?

Direct ‘transitions’ between lethal and GOF refers to mutations that are close in latent space. Examples for GOF mutations close to lethal ones in latent space are L1101E, G1097D, M1079A, E1103K, K1093F, F1084I (see Fig. S3). These GOF mutants appear to be at the boundary between GOF and lethal mutants in latent space. This may suggest that these GOF mutants manifested similar fitness values with lethal mutants, and they may be close to the viability threshold. We hypothesize that these GOF mutants may be more likely to be converted to lethality by additional mutations. An earlier study on double mutants shows that the combination of G1097D and F1084I with another GOF mutant E1103G caused lethality (Kaplan et al., 2012, PLoS Genet, 8(4):e1002627) consistent with our hypothesis. We added more explanation in the text (see page 11).

6. Fig. 1c: what the z[0] and z[1] stand for?

This is Fig. 1E in the updated Fig. 1. They are the latent space variables. We added more explanations in the caption of Fig. 1E.

7. Page 13, line 225: what's the biological outcome when GOF pheonotypes crosses the threshold for viability?

The outcome would be lethality for any enzyme that has function reduced or altered beyond the threshold required for viability. A previous study showed that some of the GOF-GOF double mutants caused lethality (Kaplan et al., 2012, PLoS Genet, 8(4):e1002627). We think this might be due to an increased GOF phenotype that may have larger combined impact in the active site dynamics compared to single mutants and result transcription defects that causes lethality. Mutants with more severe biochemical defects, either LOF or GOF, both result in severe growth defects presumably due to accumulation of transcription defects. We added a short note at page 16.

8. Page 14-15, line 253-257: It is difficult to understand their conclusions from Fig. S7. It is not clear why lethal mutants (phenotype < -1.5) have effects mostly on the TL-GTP distances while the distances between TL and BH residues are affected by mostly LOF mutants. Can the authors explicitly explain it?

For lethal mutants the heatmaps show a minimum energy for large GTP-TL distances (around 8-12 Å), while the other distances for lethal mutants are moderately changed. LOF mutants showed larger distances for GTP-TL, TL-TL and BH-TL pairs as the corresponding heatmaps cover minimum energies for larger distances for LOF phenotype. We modified the manuscript to clarify these points (see page 18) and the results are shown in Fig. S9.

9. Page 17, line 290-296: as the clustering results in a mixture of phenotypes, does that implicate that the clustering algorithm is not appropriate? How about K-center or K-medoids? My concern is that if the phenotypes are mixed within the cluster, the later analysis about the cluster center may not represent the features of the specific phenotype.

The mutant distribution on the latent space does not show a clear separation of phenotypes. However, some degree of clustering of phenotypes is visually apparent and it was captured by kmeans clustering. The clusters from the kmeans algorithm show a trend of phenotypes that the clusters II and III have more lethal and LOF mutants while cluster I has more GOF and WT-like mutants. However, we acknowledge that the distribution is very noisy, presumably because different mutants cause certain functional outcomes for a variety of different reasons. The goal here was to find the most generally applicable rules by focusing on commonalities in each cluster that relate to the mostly populated phenotypes of each cluster.

With the suggestion of the reviewer, we performed different clustering algorithms and the results are added into SI (see Fig. S19). Kmeans and K-medoids provided better separation than the other clustering methods. We did not see any significant improvement with using K-medoids over Kmeans.

10. Also, for Kmeans, the center may not lie in a real data point and how to calculate the distance for the center in this scenario?

Our rationale for selecting cluster centers comes from the concept of regularization that is applied in the VAE model. Because of regularization, latent space points between real data points should be meaningful as well. To further test this assertion, we selected the real data points that are closest to the cluster centers instead of the geometric cluster centers and re-generated the distance plots. The real points belong to 1076N (phenotype is 1.02), 1080W (phenotype is -2.14) and 1081N (phenotype is -1.85) for the clusters I, II and III, respectively. As can be seen from the figures, the results are almost identical. We added text that using the closest real points instead of the geometric data centers gives very similar results (page 23).

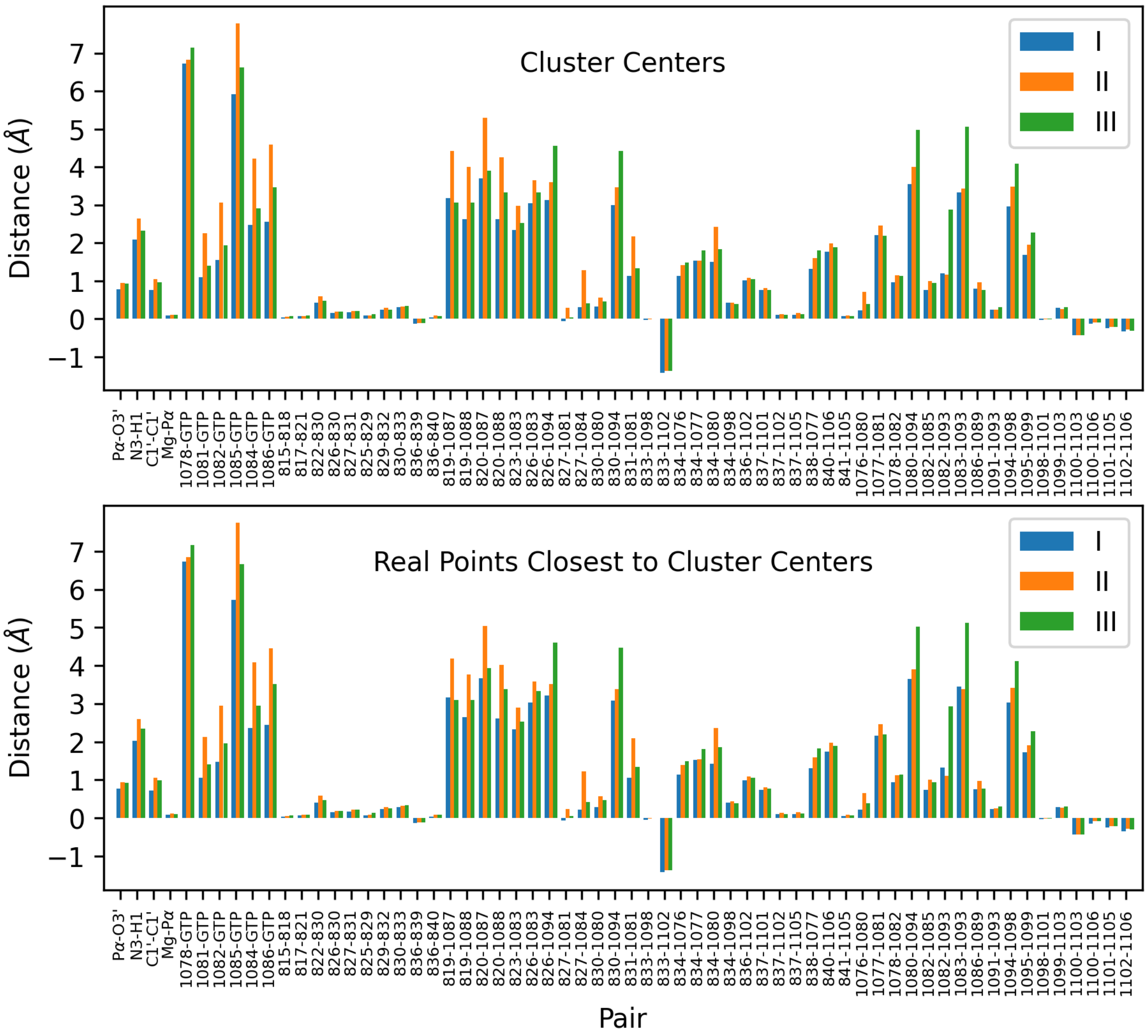

11. A summary or discussion about how to achieve a GOF phenotype by TL mutations would be helpful for the further experimental manipulation.

In this study, all the single TL mutations are covered in the experiments. Based on our ML model we then predict phenotypes for all mutations. Further experiments may focus on double mutations of TL and mutations of residues outside the TL, especially those that are in close

distance to the hydrophobic pocket that is relevant for achieving GOF phenotypes. We discussed this further in the Discussion section (see page 29).

Minor comments:
1. In the abstract, please spell out “GOF”.

The GOF abbreviation is explained in the abstract.

2. Fig. 6B: please color the y-axis in panel B by the type of interaction pair

We added color bars with an explanation for each distance type in Fig. 6B

Reviewer #2: Current ms contains studies using machine learning (ML) methods to analyze RNA polymerase (RNAP) mutants to predict mutant phenotypes from protein sequence and to probe whether molecular dynamics (MD) simulation data can improve the prediction. The RNAP mutants were generated systematically on a key structural element, the trigger loop (TL) of the RNAP, and the authors were able to demonstrate that the TL amino acid sequences can be trained to show R2~0.68 correlation with the phenotype quantities, i.e., an indication of some extent of predictability. In contrast, while the MD simulation (over tens of microseconds in aggregation) data were introduced, the current ML implementations could not really improve the phenotype prediction over that from the protein TL sequences. The work presents well intentions on bringing structural dynamics from all-atom MD to improve the ML performances, and then shows that current implementations have not been able to achieve the intended improvements yet.

It is healthy attitude to show with substantial efforts on the implementations, the performances are not necessarily as expected. On the other hand, I doubt whether the MD simulation datasets produced so far have been exhaustively analyzed to draw t conclusions or provide sufficient controls to help future improvements. Certain insights on the TL mutations to phenotypes or RNAP functions are provided. However, it was not clear whether some of insights were drawn from current datasets (upon the ML implementations), or more or less based on the authors’ prior expertise or knowledges on the RNAP system. In presenting, some of statements made in the text are not well connected with data or no figures were shown to support. Some early part of ML implementations in the absence of the MD simulation data were not sufficiently explained to facilitate learning. Detailed points are provided below for the authors’ consideration of improvements before this ms can be further considered.

1) Introduction (1st paragraph): “Proposed mechanisms for the NAC emphasize conformational changes of a highly conserved domains in the active site, present within the largest subunit of yeast Pol II, Rpb1. One of these domains is called the trigger loop (TL) and the other is the nearby bridge helix (BH).”

Have the authors considered regions beyond TL and BH within the ‘highly conserved domain or domains”, i.e., to be probed for analyses, as current TL and BH part of analyses might not contain sufficient information or notable improvements for the ML implementations?

We focused on the analysis of the TL because that is where the systematic comprehensive experimental mutation data was available. As for including additional features beyond the TL, the active site neighborhood as well as the BH are the closest structural elements and those most likely relevant for function. Elements further away could also play a role, but the number of features would increase rapidly. At the same time, the number of data points (i.e. the number of mutants) is relatively small and using a much larger number of input features than data points would likely result in overfitting and models of limited use. However, we tried using different set of data like dihedral angles of TL residues or all the pairwise distances of TL residues, but the predictions were not improved. We added comments in the text to clarify these points (page 18).

2) Continued with the above text: “The TL has open and closed conformations, which are known to be important for the nucleotide addition … Upon initial binding of the NTP, the TL closes and catalysis is promoted for substrates base-paired with the template. This results in the pre-translocation (substrate added) state, followed by TL opening together with the pyrophosphate ion (PPi) release, and subsequent or concurrent translocation”.

In the MD simulations conducted, were the TL modeled in the open or closed conformation, or both? It appears the both TL open and closed conformations are important in the nucleotide addition cycle, so catching both the TL open and closed conformations appear to be necessary.

The simulations were performed with the closed TL since this is the form that catalysis takes place. Running simulations with open TL may not be very informative since the TL in the open conformation is not supporting catalysis and many features relevant for catalysis are not apparent. We may gain more insights by simulating the transition from the open to the closed TL to see the effects of mutations to the transition path and intermediate states like has been done in a previous study (Wang et al., 2013, Biophys. J, 105, 765-775). A full analysis of the open to closed transition would be very challenging and computationally demanding for just a few mutations and probably next to impossible for the large set of mutations described here via simulations with the closed TL. Future work may focus on developing new simulation protocols that could allow such large-scale mechanistic simulation studies.

(3) Introduction (2nd paragraph): “In previous studies, a complete deletion of the TL from different species caused marked reductions in transcription rate (23, 28, 29).”

When the TL was completely deleted, were there still marginal transcription activities documented? If so, such activities in the absence of TL would be quite interesting and deserve to be included in the modeling for phenotypes.

The TL is crucial for transcription and in the absence of TL the catalysis is too slow with a 10^2-10^4-fold decrease in the rate (Yuzenkova et al., 2010, BMC Biology, 8:54; Temiakov et al.,

2005, Mol. Cell 19, 655-666) and with too many errors (Yuzenkova et al., 2010, BMC Biology, 8:54). In this study we were interested in more subtle effects of TL residues on catalysis by mutating them. Running simulations without TL would not provide us any information on specific TL residue effects and therefore would be outside the scope of this work.

(4) Continued with the above text: "On the other hand, E1103 mutations are known to cause an increased catalytic rate but with compromised fidelity"

How did the authors account for the E1103 mutations, only as 'gain of function' or GOF, or 'loss of function' or LOF, or both? Did such choices of GOF/LOF on the E1103 type of mutations (how many of such types beyond E1103) impact on the ML implementations?

Almost all mutations of E1103 showed GOF phenotypes, except E1103P that shows a LOF phenotype. There are other residues that have a majority of mutations with GOF phenotypes like L1101, G1097 and M1079. The ML models were trained to learn variations in phenotypes as a function of sequence. Because of limited data, the models had more difficulties, however, in fully predicting opposite phenotypes for certain mutations of a given residue against a background of a predominant phenotype for most mutations. It is otherwise difficult to dissect how specific data points for a specific residue (i.e. E1103) affected the overall ML model. We added more discussion on this to the manuscript (see page 14)

(5) Introduction (3rd paragraph on page 2) "To address this, we combined the data from experimental fitness scores and molecular dynamics (MD) simulations for TLs with different amino acid sequences to predict functional and structural outcomes of TL mutations using machine learning (ML) frameworks."

How were the experimentally fitness scores measured? Technically, through the ms and SI, I cannot find explanations on such basics. It is important to understand the datasets used in the analyses, so it is necessary to explain all related data measurements. To be more specific, what about the fitness scores measured for those notable mutants mentioned in the Introduction, as one may wonder whether those fitness scores were reasonably constructed in the first place.

The generation of the fitness scores follows a well-established approach that has been described in a previous paper (Qiu C et al., 2016, PLoS Genet, 12(11): e1006321). There will be an accompanying paper that will provide further details for the complete mutant matrix used here. Therefore, in this manuscript we provide only a brief description of the experimental approach, along with review-only material with additional details that previews the upcoming experimental manuscript.

(6) Results (1st paragraph) "We describe here three sets of results: 1) Based on fitness data from second generation deep mutational scanning of the Pol II TL in S. cerevisiae, we generated a model for the classification of TL mutants along a continuous phenotypic spectrum; 2) we applied ML to infer TL mutant phenotypes from TL sequence with and without structural data from MD simulations using a subset of gold standard mutants as training data; and, 3) we

extracted mechanistic principles for how different classes of TL mutations modulate Pol II function based on variational auto-encoder (VAE) models that were trained on MD simulation data." and Fig 1.

Further clarifications on the above approaches are needed. The above outlines list VAE models in the third approach. In Fig 1 to present for the first approach, VAE model was also used in Fig 1C. Are VAE techniques instead of some type of 'models'? In Fig 1C, what is the 'latent space' or what are the z[0] and z[1] data? The authors did not explain the jargons "latent space' or VAE etc., which would bring difficulties to readers who are not ML experts but still want to learn the implementations here. Besides, the fitness data in Fig 1A are shown for different conditions (x-axis) and TL residue number (y-axis), what are those different conditions, and are the fitness data measured for each TL residue but not just for each mutant species? Again, it's confusing if one wants to understand the fitness data.

We used variational auto-encoder models for both fitness and MD data. We modified the quoted sentence to clarify this. We explained z[0] and z[1] in the caption of Figure 1. The latent space is the reduced dimensional space that has the encoded properties of the input and this encoded data on the latent space could be used to generate the same properties as an output in the decoder. We added an explanation of latent space (see page 10). The fitness data provided in Figure 1A are for all the mutants (in total 589 mutants). We labeled y-axis with only the residue number to make the figure axis less crowded, since for each residue there are 19 mutants. We clarified this in the figure caption.

The experimental conditions are explained in more detail in the review-only material. A separate paper is in progress that will explain the experimental details more thoroughly. However, we briefly mention the conditions in the methods section. These conditions are phenotyping plates that fitness scores come from the allele frequencies obtained from the colonies grown in these plates. The fitness scores were obtained separately for all possible mutants of each TL residue. The fitness scores for each condition were previously associated with different phenotypes as described in a previous paper (Qiu C et al., 2016, PLoS Genet, 12(11): e1006321). Here, we introduce a continuous phenotype that is predicted from an ML model based on the fitness scores.

(7) Continued with the above text (lines 132-137 on page 7).
In the previous deep mutational scanning, how much percentiles of mutants were classified as GOL/LOF/lethal, respectively? What are the necessities or advantages of using "a continuous phenotypic spectrum" instead of doing classifications (or discretized) as GOF/LOF/lethal etc.? What is the purpose of "project the phenotypes into a two-dimensional latent space and analyze", just dimension reduction? Why only two but not say three dimension?

In the previous work by Qiu at el., they found mutations with 55 GOF, 114 LOF phenotypes and 73 of them were lethal, this study did not have the complete TL mutation library though.

A continuous phenotype was introduced to allow a more quantitative analysis, facilitate machine learning, and to capture gradually varying phenotypes between the three major classes introduced earlier as that more likely reflects the actual functional outcomes.

There are two main reasons that we applied the VAE model. First, we wanted to compare the regression model that trained against known phenotypes as a supervised learning model with the unsupervised VAE model to test whether similar phenotypes are clustered together in the reduced dimension even without supervision, suggesting that there are inherent features in the input data that give rise to distinct phenotypes. The second reason was to reduce the dimensionality to focus on overall general features and learn major distinctions between different phenotypes.

We also tested a three-dimensional latent space but found that the ML model performance is very similar with 2D and 3Dlatent spaces (Fig. S1). Therefore, the 2D space was considered to be sufficient.

We added these points to the manuscript (see page 10).

(8) Results "Fig. 2A shows the average prediction performance with good correlation (R2 = 0.68)", would the performance or correlation values change by changing parameters in ML implementations, e.g. how many mutants used for training and how many for testing.

In this study, we have a limited number of data and therefore the performance of the ML models is also limited and tuning of the parameters had minor effects on the overall performance. Following the suggestion of the reviewer we changed the sizes of the training and test sets. Decreasing the size of the training set from 100 to 80 reduces the performance significantly (R^2 values are 0.52 and 0.44 with 100 and 80 data in the training sets). Increasing the size of the training set from 100 to 120 slightly improved the performance (R^2 values are 0.52 and 0.54 with 100 and 120 data in training set). However, increasing the training set leaves fewer data points for validation. We added these results in the revised manuscript (page 13).

(9) Results (page 12) "Generally, our model predicted additive effects double mutants on phenotypes in which similar phenotypes showed an increase effect while opposite phenotypes suppressed each other".

Are those residues in mutations showing additive effects spatially separated (or distant) so that to be able to "additive"?

These models use the sequence as the only data and therefore they are insensitive to spatial information. We observed additive effects for all the double mutants we tested, essentially as a result of the ML model being trained only on single mutants Briefly, we observed increased phenotypes when both mutants have the same phenotype and suppression of phenotypes when the two mutants have the opposite phenotypes.

(10) MD simulation studies on 135 mutants: the systematical choices of mutations and over tens of microsecond simulations were impressive. The choices of relevant intramolecular distances (TL-BH etc) also appear reasonable. However, to take advantage of current simulation data, the authors might test if the performances of ML implementations vary depending on the choices of the relevant distances. If say to improve the performance is hard, one can at least test if the performance can be worse by choosing another set of intramolecular distances or even random ones. The reason to do such is to see whether there are correlations between the performance and the choice of the relevant intramolecular distances; if yes, then the performance can be expected to improve in the future once a better or an optimal set of 'distances' is obtained. Meanwhile, have the authors try to add features, e.g., dihedrals or energetics from MD to possibly improve the ML performances?

Pairwise distances and dihedral angles or energies are metrics that are expected to be correlated with each other, therefore, we assume that using one or the other would approximately provide similar results. To test different inputs from the MD data, we used TL dihedral angles as an input instead of distances and the results did not improve (R^2 values are 0.17 and 0.19 for the models with the dihedrals angles and the distances, respectively). The major conclusion here is that the 100 ns MD simulations produced data that is too noisy to be used in predictions of functional phenotypes. Therefore, using fewer input features is not likely to give much insight. Instead, one probably needs much more extensive MD data over much longer time scales to better correlate MD-extracted features with functional outcomes.

In addition, since the authors suggest that longer or microsecond simulations can possibly improve the performance of the ML implementations, then the authors can test using current MD simulation data set (e.g. 100 ns each trajectory), if a choice of short or non-equilibrated simulations (e.g. 0-50 ns trajectory) or medium stage (e.g. 30-80 ns) impact on the performances, i.e., to make the performances worse. If these impacts are notable, then one has better reasons to believe that longer (>>100 ns) or better equilibrated trajectories has the potential to improve.

We performed the ML calculations using the distances from 50-100 ns portion of the simulations. The results did not change much when using 0-50 ns (R^2 values are 0.18 and 0.19 for the models using 0-50 ns and 50-100, respectively). This may be expected if it is in fact necessary to reach microseconds or even longer time scales to use MD data more effectively to predict functional outcomes.

(11) Fig 4: the authors well list five groups of distances (A) and (B), but in (C), only show the heatmap for the fully averaged distances. It would be much more informative to show five heatmaps for respective five groups of distances, so that the authors can really point out some of the data structures in the figure to make reasonings, while currently the authors often make statements on structural contributions without referring to any data.

We showed the heatmaps for the five groups separately in Figure S9. We tried to clarify the statements that are not clear and explained them in the following corresponding comments.

(12) Fig 5: MD wo/w attention. What is ‘attention’, pls explain for readers lack of ML knowledges. In VAE model presented on page 17 “… we also added an attention layer both on the encoder and decoder sides”, pls explain why or what the purpose of adding an attention layer.

Attention is a machine learning framework that learns to focus on important parts of the input as a function of other input features. We applied attention to account for the effects of mutations to different sets of distances, e.g. some mutants affect the GTP-TL distances, while others affect the BH-TL distances. We added more explanation on the purpose of attention layer in the manuscript (pages 21 and 36). We also added a figure that shows the separation of mutants on VAE latent spaces generated with and without attention showing that the model with attention layers provides a better separation of the phenotypes (see Fig. S17).

(13) Fig 6 on VAE models: Fig 6A, what are the data axis Z[0] and Z[1] represent, or what the matric used to conduct the clustering/K-means? Since different phenotypes scatter in different clusters, no much statistics are provided as well, it is not obvious how one can learn from the clustering in this latent space.

We explained z[0] and z[1] in the caption of Figure 6. Essentially these are arbitrary, normalized generalized coordinates that result from the dimensionality reduction into the latent space by the VAE method. The Kmeans clustering was performed using the latent space coordinates of the mutants, therefore it is the geometrical clustering of mutants in the latent space. Then we could generate representative distances for each cluster to classify the effects of mutations on structural data and make connections with the functional phenotypes, although the phenotypes are not well separated in the latent space. We modified the sentence in page 21 to clarify the kmeans clustering step. We also note that we provided a statistical analysis of how phenotypes are separated within the clusters in Table S2.

Fig 6B, is it better to show cluster I/II/III data separately (not mixed) to reveal some data structure patterns? It’s also more informative to show for different distance groups (e.g. those five groups in Fig 4AB). Again, the authors should show data explicitly from which they drew conclusions, not just provide conclusions/statements without showing the data.

We are showing the distances for the cluster centers in the same figure (Figure 6B) to make the comparison more easily between the clusters. The mentioned conclusions/statements are the conclusions of these distances that we explained below and we modified the manuscript to clarify those points.

We added the heatmaps for the five distance groups (Fig S9) and we modified Fig 6B to emphasize those groups in different colors.

For example, where are the data that support statements below:
“longer intra-molecular distances for key residues around the TL for LOF/lethal mutants”;

The cluster centers of the clusters II and III showed overall larger distances (for the entire distance set in Figure 6B) than the cluster I. Since the cluster II and III are dominated by LOF/lethal mutants, we concluded that LOF/lethal mutants have more effect on distances.

“Cluster II features larger distances between TL residues and GTP compared to the other clusters”;

This is also a conclusion of Figure 6B that shows larger distances for most of the TL-GTP pairs for the center of Cluster II compared to the centers of clusters I and III. We also provided some of the members that features large distances for TL-GTP pairs in parentheses. All the distance data is provided in the Supplementary Spreadsheet II.

“Cluster III also features larger distances 321 between TL and GTP for some mutants”;

This result is from selected mutants in Cluster III provided in the parentheses. We observed larger distances between GTP and H1085/L1081 for some of the members of cluster III, which shows that cluster centers are not reflecting all the mutants in the clusters, that there could be some deviations in distances.

“cluster III mostly features increased distances for BH-TL pairs”

This is a conclusion from Figure 6B, that shows larger distances between V1094 and K830/D826 for Cluster III compared to Clusters I and II.

“Some of the TL-TL distances for the residues that are close to V1094 are also increased for the cluster III”

Again, Figure 6B shows that the distances between V1094 and T1080, V1094 and V1098, K1093 and T1083, are larger for the cluster III compared to clusters I and II.

Fig 6F-H showing “M1079N and E1103G in the clusters III” and “Both M1079N and E1103G lead to disruptions of the hydrophobic pocket although the mechanism may be slightly different”, and concludes “results support the hypothesis that the primary mechanism for GOF phenotypes is via the direct or indirect disruption of the hydrophobic pocket formed by TL residues near the BH as suggested by previous studies (30, 32).”

How many GOFs or how much percentile show the “disruptions of the hydrophobic pocket”? It is not clear whether and how the VAE model and clustering data help to bring the insight on “GOF phenotypes is via the direct or indirect disruption of the hydrophobic pocket formed by TL residues near the BH”.

To understand the effects of mutants to the hydrophobic pocket, we showed last snapshots of some of the selected mutants in Fig 6F-G. These results are coming from the direct visualization of the structures obtained from simulations.

In order to analyze the hydrophobic pocket quantitatively, we calculated the hydrophobic contacts in this region for all the mutants and the surface area made by the residues around the pocket (see Fig. S13). We found similar mean values and not significantly different distributions. However, some of the GOF mutants showed smaller number of contact and larger surface area compared to other phenotypes suggesting a disruption of hydrophobic pocket by these GOF mutants. We added more discussion about this analysis in page 26. The analysis results are shown in Fig. S13, and the data is provided in Supplementary Sheet 2.

(14) Conclusions (the last paragraph): I'm wondering about how the "VAE models developed based on MD still could provide mechanistic insights into the potential structural basis of lethal, LOF, and GOF mutants": are VAE models basically doing the dimension reduction?

MD simulations provide structural data of the mutants, and this large amount of structural data is difficult to be understood in general terms without dimensionality reduction. The VAE model based on the MD data makes it possible to identify common structural features for different phenotypes by mapping latent space variable to input features via the decoder part of the VAE architecture.

Again, I'm also still wondering about where to find the VAE related dataset (or figures) to reach conclusions in the text: (i)"lethal and more serious LOF phenotypes correlate with mutations that directly increase the distance between key TL residues and the GTP in the active site"; (ii) "Weaker LOF phenotypes for the examined mutants appear to be related more to disruptions in the TL-BH interaction"; (iii) "a substantial fraction of GOF phenotypes appear to result largely from a disruption of a hydrophobic pocket near the TL"; (iv)"In most of these GOF mutants, we observed increased BH-TL distances especially for the cases when the mutants located close to BH".

(i) and (ii): We observed two clusters (II and III) that have predominantly LOF and lethal mutants. Distance analysis for cluster centers in Fig 6B shows that cluster II shows larger distances between GTP and TL residues while cluster III shows larger distances between some of the TL-BH residues. Fig. S11 also support this for selected individual mutants from these clusters. We modified these sentences to clarify the points.

(iii) We modified this sentence; the disruption of hydrophobic pocket was observed for some of the GOF mutants.

(iv) We corrected this sentence to reflect that a subset of GOF mutants have larger BH-TL distances that is supported by Figs. S12.

Reviewer #3: The authors of the manuscript engage is the challenging task of trying to correlate genetic fitness scores with sequence-based machine learning and structural insights from molecular dynamics.
A general issue with the manuscript is that it presents a rather "yeast-centric" perspective. Given the high degree of evolutionary conservation of RNAP active site function across the evolutionary spectrum, the authors do themselves (in my view) a disservice by not emphasizing sufficiently this universality and not taking a broader view in their Introduction and Discussion sections. Inclusion of insights gained from mutations in archaeal and bacterial Trigger Loop mutations would make the manuscript more relevant to a wider readership.
This paper is going to be very challenging for "average" molecular biologists to read (who are the most likely target audience to benefit from the biological take-away messages), so most of the recommendations for improvements given below are aimed at encouraging the authors to explain some of the key biological and computational assumptions and concepts in more detail.
Overall, the authors succeeded only partially in their ambitious goal. This is, however, not meant as a major criticism, but rather reflects the complexity of the topic and the technical shortcomings in obtaining the right biological data sets and limitations of computational resources and approaches. The manuscript should be seen as a brave attempt to tackle a fundamentally important question and gaining insights that are worth publishing.

======================
Specific Recommendations:

Abstract:
Line 45: GOF abbreviation not defined

We added the definition of GOF in the abstract.
Author Summary:
Line 58, 59: The sentence concerning effects on the hydrophobic interactions is very vague. Maybe more detail/specific information to sharpen up the argument?

We modified this sentence for further clarification.

Introduction:
The description in line 68 applies to RNAPs from all cellular organisms. To apparently limit the description to "yeast Pol II" is too narrow. The universality of the NAC in all cellular RNAPs should be emphasized. A new figure, showing the sequence alignment of TL sequences from a variety of archaeal, bacterial, and eukaryotic species, would provide an excellent background to visualize the relationship of residues in yeast RNAP discussed in the manuscript with the degree of evolutionary conservation. Such a sequence alignment is even essential for the reader to get an impression concerning the fundamental question addressed in the manuscript: how do functional TL phenotypes resulting from residue variations manifest themselves on a structural and dynamic level? Some residues are evolutionarily highly conserved, others less so, thus already providing at least partial insight into a potential answer.

Here we wanted to emphasize that this current work is specifically on a yeast structure. We agree that this description applies to all organisms. Following the suggestion, we added sub-figures in Figure 1 (Fig. 1A and 1B) that show sequence conservation of TL residues and the TL structure colored based on this conservation. We modified the text accordingly (pages 8-9).

Results:
Line 133, 135: What is meant by “indeterminate” phenotype? How is this defined? What are the reasons for combining this with the wildtype data?

The indeterminate phenotype was used for mutants that show slight phenotypes for certain conditions and not showing any strong phenotypes in any conditions; therefore, they can be classified as near-WT phenotype. We added a short explanation about it in page 8. More details can be found in the paper by Kaplan Lab (Qiu C et al., 2016, PLoS Genet, 12(11): e1006321).

In Figure 1, it does not mention what these mutants actually are! A brief description is absolutely essential here. Also, what is the rationale for the different assay conditions? What are they supposed to test? In which parts of RNAPII are the mutations located that are tested in combination with the TL mutants? More information is necessary here.

Figure 1 shows all the single mutations for TL residues. We added more information in the caption for clarification.

The different conditions are corresponding to the plates for phenotyping. Each assay demonstrates different phenotypical effects as explained in the previous paper (Qiu C et al., 2016, PLoS Genet, 12(11): e1006321). This work, along with earlier studies (Kaplan et al., 2012, PLoS Genet, 8(4):e1002627; Braberg et al , 2013, Cell 154, 775-788) demonstrated that biochemical behavior in vitro was strongly predicted by phenotypic profiles in vivo. For example, SC-Lys plate utilizes the suppressor of Ty phenotype (Spt-) wherein WT cells cannot grow but some mutations of Pol II allow the expression of the reporter allele and these mutations are mostly found to be related to the GOF phenotypes. Having multiple kinds of plates can help to cover a larger set of phenotypical outcomes of the mutation library. More information about the effects of different conditions can be found in the previous paper (Qiu C et al., 2016, PLoS Genet, 12(11): e1006321). There is also an experimental paper in progress that explains generation of the complete mutant library. In order to facilitate the review of the present paper, additional details about the experimental procedure were provided as “review-only” material.

There is obviously a rather substantial conceptual problem with the phenotypic range data set: averaging results from a range of assays based on potentially mechanistically functions may hide some interesting effects of some of the mutants under certain conditions. The authors mention that this set-up allows them to distinguish between LOF and GOF mutants, but this seems a rather modest achievement compared to the potential richness of mechanistic differences hidden in the primary data under the various assay conditions. Rather than combining the data into an averaged fitness score, the authors should consider running the ML model for each of the experimental conditions independently to see if some of the experimental conditions identify

particular mutant properties that gets lost in the average score. Could this averaging effect also explain why extreme outcomes (Line 195, 196) are not predicted reliably by the model because some of the data that would help to predict such outcomes more reliably got “submerged” in the phenotypic score used for the training?

In this work, we tried to look at the big picture of how we classify mutants using a large set of fitness conditions. A more detailed explanation of these conditions was provided for the first generation of mutant library in the previous paper (Qiu C et al., 2016, PLoS Genet, 12(11): e1006321) and will be discussed in the upcoming experimental paper. This present manuscript focuses on commonalities in the functional phenotypes and their structural determinants. A more detailed analysis of further insights that may be derived from comparing different assays is beyond the scope of this work. Difficulties in predicting the extreme outcomes using sequence model could be more related to the data size of the training sets. Small data sets may not provide enough information about the extreme outcomes; therefore, the models cannot learn those outcomes during the training.

Line 141: “we trained a neural network model” is rather unspecific; even in the main text, the basic architecture (256, 128 and 64 nodes, and 128 and 64 nodes connected to the output layer via an attention layer; as described in Materials and Methods) should be mentioned, especially considering that this manuscript is under consideration for a computational journal. Are there any reasons for choosing these particular architectures? Why was a self-attention framework considered? How do these two architectures compare? Where any other architectures tested? How long was the model trained? Was there any evidence of overfitting and how was this possibility excluded?

We added explanations of the architectures of the neural networks in the results sections as well. The specific details for each model, like the number of epochs or the loss functions that we used, are provided now in Table S1. We tried different architectures that gave either similar or worse results, and we describe here only the final architectures. An attention layer was used to account for the different effects of the mutations on different part of the structure. We added more explanation for the attention layer in the manuscript (pages 21 and 36). We showed the VAE results with and without attention in Fig S17. In VAE models we tried to project the fitness scores on to the reduced dimensional space; therefore, we used all the data for this model without separating it into training and test sets. We note that in VAE models there is an additional regularization term (Kullback–Leibler divergence term) that minimizes the difference between the latent space distribution and normal distribution. This helps avoiding overfitting of the latent space and providing meaningful latent space points. The VAE model with MD data provided almost identical output distances for cluster centers with respect to closest real points to the cluster centers (see the Figure in response to Reviewer 1), which could be one evidence that the overfitting was avoided during the learning of latent space distribution. We added a note in page 23. In the predictive models, on the other hand, we tried to minimize overfitting by applying cross-validation over different random training/test sets and early-stopping the training to obtain the minimum test loss.

Figure 2 provides a great overview of the prediction, but once again I miss an evolutionary sequence alignment at this point. Residues 1077, 1078 and 1080-1088 are predicted to be rather sensitive to mutation, whereas positions 1090-1104 are either insensitive or prone to GOF phenotypes. How does this correlate with the evolutionary conservation? Also, for readers less familiar with TL structure, showing a simple spatial model of the TL structure with at least some of the residue positions marked would be helpful.

The modified Figure 1 has the sequence alignment of TL and it is structure in yeast. We added more discussion about the mutant phenotypes and their evolutionary connections. Overall, we observed that the residues between 1077-1088 are more conserved and the mutations of these residues have mostly lethal and LOF phenotypes.

Line 214: What is the rationale for attempting double predictions - spell out the reasons for clarity.

The ML models were trained on single mutations and we were curious to see whether they could nevertheless predict the outcomes of double mutations. If successful, the practical purpose would be coverage of a much larger mutation space than what could be achieved experimentally. From a functional point of view, double mutants may be especially interesting when reversing functional outcomes of single mutations.

We added an explanation for the purpose of testing the models for double mutant predictions (page 15).

Line 221: "This additive prediction is consistent with previous studies" is too enigmatic; not everybody has the time to look up the reference provided. Provide a brief explanation how this data fits previous results.

We added more explanations about the results of previous studies.

Line 230, 231: it is a pity that this potentially very powerful prediction has not been tested experimentally yet. A successful outcome would strengthen the value of the model presented in this manuscript considerably!

We are planning to continue working on double mutations in future work. The specific focus of this work is on computational analysis and hypothesis generation based on already existing experimental data.

Line 236: Once again, I consider a brief description of some of the thinking concerning the MD simulation set-up to be essential at this point. Why was a simulation of the whole RNAPII structure (PDB 2E2H) considered to be essential? Longer simulations on a smaller structure only

encompassing the elements of the active site could have been a more productive use of computing resources. Given that the authors simulated the whole RNAPII molecule, did they find any evidence that any of the TL mutants had any long-range distance effects on structures outside the active site?

We simulated the complete Pol II structure since it has a complex arrangement of subunits, and it is difficult to decide which parts of the complex should be included. Both Rpb1 and Rpb2 span the active site and removing the parts outside the active site would create instabilities in the structure and potentially provide unreliable results and/or introduces biases if restraints were applied to maintain stability in a truncated structure. We actually tried simulations with truncated Pol II structures some time ago but decided that the trade-offs in simulation accuracy were not worth the computational savings. Therefore, we performed the simulations on the complex with 10 subunits. For the analysis, we only focused on the active site, or more precisely, the vicinity of the TL mutations characterized experimentally. However, we plan to further analyze the trajectories to address additional questions in the future.

Why was the system only neutralized and not simulated under a more "physiological" (150 mM NaCl) condition? Considering that the NTP is highly charged and that TL-NTP interactions could be affected by ionic interactions, this is a somewhat "courageous" way of setting up the simulations.

We simulated neutralized systems that have around 60 Na^+ ions to act as counterions for the negatively charged nucleic acids. We did not use a specific ion concentration since it was difficult to decide a concentration that would best account for varying experimental conditions under which the fitness scores were generated. While adding more ions to achieve finite salt concentrations could slightly change the results, the presence of counterions does allow for charge screening of the NTP and other charged moieties. We also note that in addition to the counterions, Two Mg^{2+} ions were present in the active site.

Overall, it seems clear that the MD data - despite the huge effort obtaining it - is not very informative. I agree, however, with the authors' decision to include the data to show the current limitations on these approaches. Obviously, it would have been great if it had worked better (and longer simulations on reduced systems, as suggested earlier, could help).
Line 283: A brief and more general explanation of VAE models, and why this approach was chosen in this instance, would be very helpful. I do not think that biologists reading this manuscript would be able to understand the reasons given in Lines 285- 288.

We added more explanations for our purpose to use VAE model (page 21).
