# Supplementary material for: Characterization of RNA polymerase II trigger loop mutations using molecular dynamics simulations and machine learning
Source: PLoS Comput Biol. 2023 Mar 22;19(3):e1010999. doi: 10.1371/journal.pcbi.1010999 (PMC10069792; doi:10.1371/journal.pcbi.1010999)
Supplement: Attachment — Submitted filename: response_reviewer_comments.pdf [file pcbi.1010999.s032.pdf]

Reviewer's Responses to Questions

*Comments to the Authors:
Please note here if the review is uploaded as an attachment.*

Reviewer #1: The authors have appropriately addressed all my comments.

Reviewer #2: The authors have made some efforts and partially addressed my concerns. Some of points left for the authors' further considerations are listed:

Point 2): the authors only modeled the TL closed but not the TL open conformation: This study is to address for full RNAP elongation function but not only the catalysis step. The TL open to closed transition is highly important for the elongation cycle as it is potentially a rate-limiting step and a dominant conformational change of the RNAP II. Hence, ignoring the TL open conformation is not well justified for the purpose of this study.

Considering that the computational cost is high already for current implementations, the authors should at least make efforts to explicitly address this issue in the ms and clarify the limitation of this work. A better way, since the lab had previously studied the TL open to close transition, as being claimed (Wang et al., 2013, Biophys. J), the authors can consider including some of previous datasets to make improved analyses or perspectives to current work.

We thank the reviewer for appreciating the computational cost of simulating transition of open to closed TL for the mutant library we studied here. This is beyond the scope of the current manuscript and will be a future direction. With the suggestion of the reviewer, we included additional analysis on the open and closed TL based on the dataset from the earlier published simulations and further discussed the data of transition from open to closed TL (Wang et al., 2015, Nucleic Acids Res., 43, 1133–1146; Wang et al., 2013, Biophys. J. 105, 767–775). The analysis results are shown in Fig S16 and the additional discussion on open/closed TL and the limitations of simulating the transition for the mutant library is provided at page 29-30.

We analyzed the distances for the pairs that were used in our machine learning analysis, for the open and closed TL simulations of WT Pol II. Open TL manifested larger distances especially between GTP-TL residues and BH-TL residues (see Fig S16). This result suggests that the distances we used in machine learning analysis are mostly relevant to the closed TL. We note that the distances that are larger in open TL also increased in LOF/lethal mutants (Fig 6B) suggesting that LOF/Lethal mutants destabilize the closed TL.

On the other hand, some of the distances between TL-TL or TL-BH residues appeared to be smaller for the open TL or for the transition states between the open and closed TL states (see Fig. 3B and Fig. S12 in Wang et al., 2013, Biophys. J.). The function of such TL residues may be related to the open state and therefore the mutant simulations we performed for these residues on the closed state may not provide any insightful structural outcomes. Open TL simulations or simulating the transition for these mutants may be necessary in future work to obtain mechanistic understanding of their phenotypes. Examples of

such TL residues include E1103 and K1093, both showed overall smaller distances at open TL state (see Fig. 3B in Wang et al., 2013, Biophys. J.).

Point 3): the request is not asking the authors to run simulations on mutants without TL, but to clarify an interesting issue why “deletion of the TL from different species caused marked reductions in transcription rate” but not fully abolished the transcription activities. The clarifications provided by the authors (with data and the reference) are helpful to be included into the ms.

We included the clarifications about quantitative effects of TL on transcription rate and fidelity in the manuscript and provided the references (pages 4-5).

Point 5): In regard to the fitness scores which serve for the basis of current analyses: the authors provided a ms in accompany with technical details; the authors also quote the reference of Qiu C et al 2016 work and claimed a brief description of the experimental approach in the ms.

Nevertheless, it’s hard to get a brief message on the basic rationale on how to define such a fitness score. If I missed it, authors may consider highlighting the sentences, or providing a couple of sentences to summarize the main messages according to their own understanding. It’s not for technical details but for intuitive understanding, as rationalizing the fitness score serves for somehow a premise of current study.

We have added the following summary of fitness to methods to allow an explicit understanding of what fitness means:

Briefly, a library of mutants is grown under different experimental conditions. These conditions detect growth changes of mutants relative to WT (phenotypes) that are predictive of Pol II biochemical defects (32). Phenotypes are expressed as “fitness” scores that represent the log2 allele frequency changes of individual mutants within the library pool over time for the series of experimental stress conditions relative to a control growth condition relative to allele frequency changes of a WT allele. Allele frequencies are determined by deep sequencing of variant pools after growth relative to starting or control conditions. Sequencing allows quantitative determination of each allele’s frequency in the library. A mutant that grows worse than WT for a particular stress condition relative to control will have a negative fitness score whereas a mutant that grows better than WT for a particular stress condition will have a positive fitness score.

Point 9): It is true that the prediction does not rely on the spatial information. Since the authors have access to the 3D structure of the RNAP II, they can simply check the structure. Note that the request is not request the authors to re-run anything but to check for their own data that are potentially more interesting than they thought: to find from the 3D structure of RNAP II on whether those mutations show additive properties are those spatially separated in the 3D locations.

With the suggestion of the reviewer, we analyzed the relationship between the additivity of phenotypes and the spatial distance between the mutation sites for the double mutants. In Fig S9A, we compared predicted phenotypes for double mutants and their additive phenotypes, which is the sum of the phenotypes of single mutants of double mutant sites. Predicted and additive phenotypes were highly correlated (R2=0.9991, MSE=0.29) suggesting that all the double mutants were additive. Fig S9B shows a plot of the differences between the predicted and additive phenotypes vs. the distances between the mutation sites. Fig S9B provides three main results: 1) predicted phenotypes are higher than additive phenotypes, 2) predicted phenotypes are closer to additive phenotypes for GOF mutants compared to LOF/lethal mutants, 3) there is a clear tendency for obtaining a better agreement between the predicted and additive phenotypes for the mutant sites that are spatially in close distance. We added more discussion about this analysis to the manuscript (see page 16).

Point 10): If the authors tested the different MD data inputs, e.g., TL dihedrals instead of distances, then such tests and results should be included in the ms (indicate where if included already).

Besides, the authors did not get the point of the quest: Using less features is NOT to gain insight but serve for control. If one shows that fewer input features bring worse results, it is equivalent to show current input (with more features) is better than using fewer. Tests on correlation between the #input features with performance score/index can help to determine how much is enough.

We included the results of additional predictive machine learning models using a set of different inputs from MD simulations to the SI (Fig S11) and added more discussion to the manuscript (page 20). The models include the original model with the five sets of distances, dihedral angles, distances and dihedral angles together and five additional models that at each model one set of distances was excluded. Fig S11 shows that the R^2 correlations were not changing much by using different MD inputs or using smaller number of distance data. This result suggests that MD data (either the pairwise distances near the active site or dihedral angles of TL residues) was not successfully predicting phenotypes.

Reviewer #3: The manuscript has been clarified in many of the areas that all three referees highlighted. Nevertheless, it remains a difficult and somewhat unsatisfactory manuscript because the author's response has been rather minimal and several of the criticisms were only addressed in a superficial manner. Especially the descriptions and explanations of key data sets remain problematic for the reader because the authors keep essential information back to publish them separately somewhere else. Full knowledge and understanding of the meaning of these data sets is, however, critical to appreciate the results presented in the current manuscript. Under these circumstances, I wonder why the authors did not wait to publish the experimental data first and possibly afterwards describe the ML-analysis described here.

We consider the experimental and computational studies as two separate projects with different goals. The original conception for computational work was to use the published 2016 data. In the interim, second-generation data were generated and gave much greater coverage of the single mutant space, so

the data are of higher quality and greater depth. These data were generated in essentially the same fashion as published data, but they allow a much deeper analysis of overall mutant space and genetic interactions among positions (double and higher order mutants).

A preprint of the full analysis of the experimental results has been submitted to bioRxiv: https://doi.org/10.1101/2023.02.27.530048 This work is now under peer review.

We believe that the publication of those data will not alter conceptually the results obtained in the current manuscript.

In response to reviewer 2, we have added an explicit description of fitness that follows the cited published work. Slight differences in experimental protocol from published are described in the methods currently. Additional details can be found in the preprint.

Combined with the fact that the authors succeeded only partially in their ambitious bioinformatics goals, my overall impression is therefore that the paper, even after the revision, has not really succeeded in providing clear, novel insights into an important biochemical mechanism. Although sophisticated in computational terms, the goal is overambitious and probably not achievable in a satisfactory way using the currently available methodology.
Although I would not have any issues for the paper to be published in its somewhat improved format, I do not see any strong reasons in favor of publication either.

Thank you for the comment.
